# Cleavage of a Neuroinvasive Human Respiratory Virus Spike Glycoprotein by Proprotein Convertases Modulates Neurovirulence and Virus Spread within the Central Nervous System

**DOI:** 10.1371/journal.ppat.1005261

**Published:** 2015-11-06

**Authors:** Alain Le Coupanec, Marc Desforges, Mathieu Meessen-Pinard, Mathieu Dubé, Robert Day, Nabil G. Seidah, Pierre J. Talbot

**Affiliations:** 1 Laboratory of Neuroimmunovirology, INRS-Institut Armand-Frappier, Université du Québec, Laval, Québec, Canada; 2 Institut de Pharmacologie de Sherbrooke, Faculté de Médecine et Sciences de la Santé, Université de Sherbrooke, Sherbrooke, Québec, Canada; 3 Laboratory of Biochemical Neuroendocrinology, Clinical Research Institute of Montreal, Montréal, Québec, Canada; Thomas Jefferson University, UNITED STATES

## Abstract

Human coronaviruses (HCoV) are respiratory pathogens that may be associated with the development of neurological diseases, in view of their neuroinvasive and neurotropic properties. The viral spike (S) glycoprotein is a major virulence factor for several coronavirus species, including the OC43 strain of HCoV (HCoV-OC43). In an attempt to study the role of this protein in virus spread within the central nervous system (CNS) and neurovirulence, as well as to identify amino acid residues important for such functions, we compared the sequence of the S gene found in the laboratory reference strain HCoV-OC43 ATCC VR-759 to S sequences of viruses detected in clinical isolates from the human respiratory tract. We identified one predominant mutation at amino acid 758 (from RRSR↓ **G**_758_ to RRSR↓**R**_758_), which introduces a putative furin-like cleavage (↓) site. Using a molecular cDNA infectious clone to generate a corresponding recombinant virus, we show for the first time that such point mutation in the HCoV-OC43 S glycoprotein creates a functional cleavage site between the S1 and S2 portions of the S protein. While the corresponding recombinant virus retained its neuroinvasive properties, this mutation led to decreased neurovirulence while potentially modifying the mode of virus spread, likely leading to a limited dissemination within the CNS. Taken together, these results are consistent with the adaptation of HCoV-OC43 to the CNS environment, resulting from the selection of quasi-species harboring mutations that lead to amino acid changes in viral genes, like the S gene in HCoV-OC43, which may contribute to a more efficient establishment of a less pathogenic but persistent CNS infection. This adaptative mechanism could potentially be associated with human encephalitis or other neurological degenerative pathologies.

## Author Summary

Human coronaviruses (HCoV) are respiratory pathogens involved in a sizable proportion of common colds. They have over the years been associated with the development of neurological diseases, given their demonstrated neuroinvasive and neurotropic properties. The viral spike (S) glycoprotein appears to be associated with these neurologic features and is a major factor of virulence for several coronavirus species, including HCoV-OC43. To further characterize the role of this protein in neurovirulence and virus spread within the CNS, we sought to identify amino acid residues that may be important for this function. Our data revealed that one of them, G758R, introduces a functional furin-like cleavage site in the S protein (RRSR↓R_758_). This change in S protein mostly impacts neurovirulence, which seems associated with a modified viral dissemination, without significantly affecting its neuroinvasive capacity. This mutation, found in all characterized contemporary human clinical respiratory isolates, underlines previous findings that naturally existing field isolates of HCoV-OC43 variants still possess the capacity to invade the CNS where they could eventually adapt and establish a persistent human CNS infection, a mechanism potentially associated with human encephalitis or neurodegenerative pathologies of unknown etiologies.

## Introduction

Human coronaviruses (HCoV) are enveloped positive-stranded RNA viruses belonging to the family *Coronaviridae* in the order *Nidovirales* and are mostly responsible for upper respiratory tract infections [1]. Being opportunistic pathogens, they have also been associated with other more serious human pathologies, such as pneumonia and bronchiolitis, and even meningitis [2–4] in more vulnerable populations. Moreover, at least HCoV-229E and HCoV-OC43 are naturally neuroinvasive and neurotropic in humans [5]. Indeed, we have previously reported that HCoV can infect and persist in human neural cells [6–8], and in human brains [9]. Moreover, the OC43 strain (HCoV-OC43) induces encephalitis in susceptible mice, with neurons being the main target of infection [10, 11].

Enveloped viruses use different types of proteins to induce fusion of the host-cell membrane to their own in order to initiate infection. For coronaviruses, the spike (S) protein is responsible for cell entry [12], and was shown to be a major factor of virulence in the central nervous system (CNS) for several coronavirus species, including HCoV-OC43. We previously reported that persistent HCoV-OC43 infections of human neural cell lines led to the appearance of predominant point mutations in the putative receptor-binding domain of the S glycoprotein gene [13] and that these mutations were sufficient to significantly increase neurovirulence and modify neuropathology in BALB/c mice [14].

In order to identify amino acid residues in the S glycoprotein that are involved in viral spread within the CNS, we compared the sequence of the gene encoding the viral S protein in the laboratory reference strain HCoV-OC43 (ATCC VR-759) with sequences of the S gene in viruses detected in clinical isolates from sputum of upper and lower respiratory tract of seven children, aged 3 to 36 months, admitted to the University Hospital of Caen, France, in 2003 [15], as well as with all S protein sequences found in the NCBI data bank. This characterization led to the identification of predominant mutations, including one at the amino acid Gly_758_, which introduces a putative furin-like protease cleavage site RRSR↓R_758_ in the viral S protein [16].

Several class 1 viral fusion proteins, such as the coronavirus S protein, are proteolytically processed during infection of the host cell, a mechanism that is often essential for the initiation of infection of receptor-bearing cells, tissue tropism and in eventual pathogenesis [17–20]. Moreover, its cleavage by different types of host proteases, including furin-like proteases designated proprotein convertases (PCs) that cleave at paired basic residues [20] are involved in various steps of coronavirus infection [21–23].

In the present study, we show for the first time, that while the S glycoprotein of the laboratory reference strain HCoV-OC43 ATCC VR-759 is not cleaved by host cell proteases, the sequences of more than 60 clinical isolates reveal a common G758R resulting from a single nucleotide polymorphism (SNP) in the S gene. This creates a functional PC-cleavage site between the S1 and S2 portions of the viral S glycoprotein, thereby modulating viral spread and neurovirulence in susceptible mice, without affecting the neuroinvasive capacities of the virus or its infectivity (capacity to infect) of a neuronal cell line. These results, which suggest that PC-cleavage can be dispensable for efficient infection by HCoV-OC43, appear surprising compared to other coronaviruses, for which S protein cleavage is required for efficient virus infection [21, 23, 24]. Importantly, our results may help to better characterize the possible adaptation of HCoV-OC43 to the CNS environment, which, in the end, results in a decreased neurovirulence potentially associated to a modified spreading and a more efficient mechanism for the establishment of a persistent infection in human CNS, a phenomenon that could influence the severity of human viral encephalitis or exacerbate neurological degenerative pathologies of unknown etiologies.

## Results

### Both rOC/ATCC and rOC/S_G758R_ variants are neuroinvasive and neurovirulent

**Fig 1:**
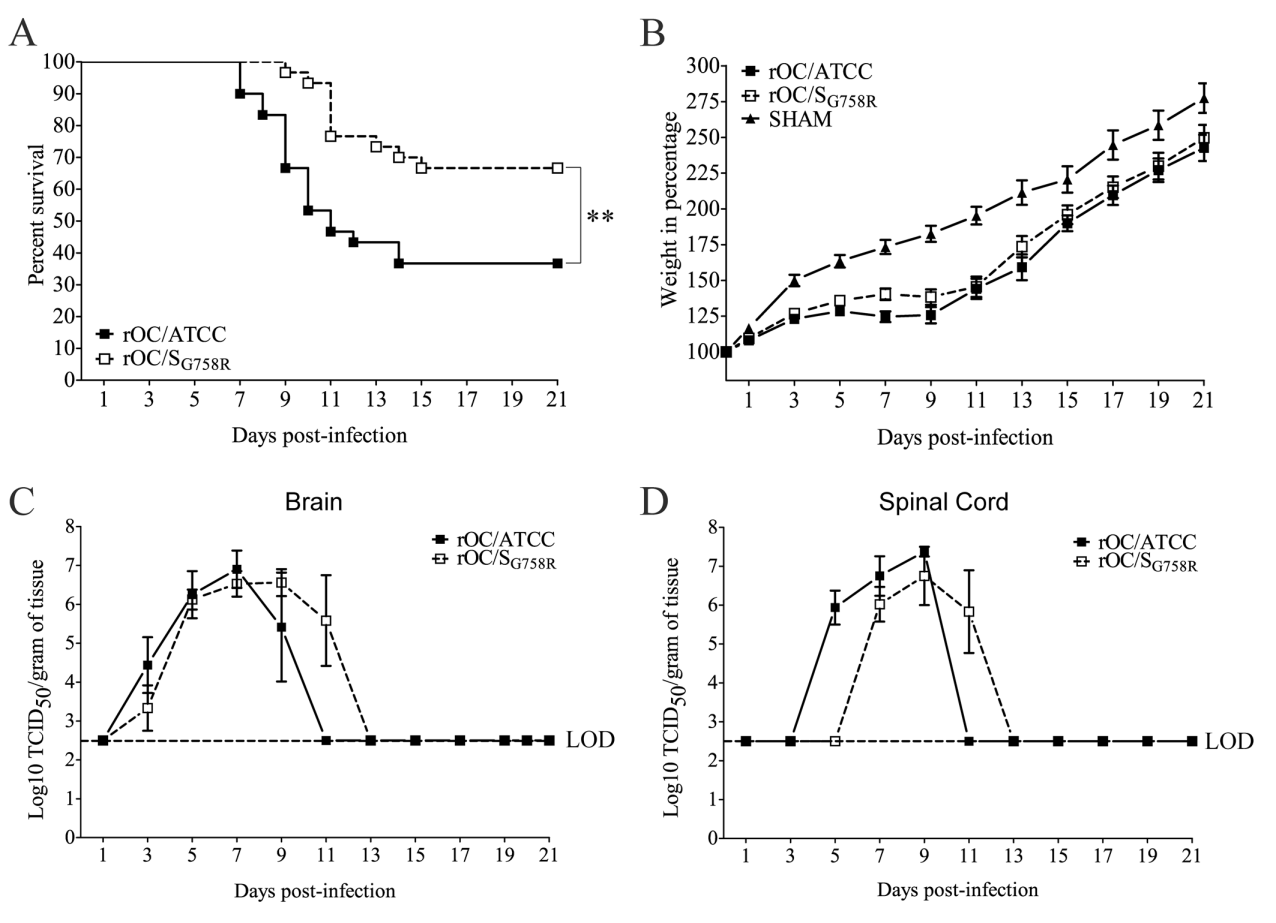
Both variants rOC/ATCC and rOC/S_G758R_ are neuroinvasive and neurovirulent in 10 day-old BALB/c mice infected by the intranasal route. 10-day old BALB/c mice received 10^3.25^ TCID_50_/10μL of rOC/ATCC or rOC/S_G758R_ or PBS by the IN route. (A) Survival curves of mice in percentage (%) during 21 days post-infection. Difference between the two variants was significant (** P≤0.01) (B) Surviving BALB/c mice were weighed every 2 days after infection during 21 dpi to estimate weight variations. The weight variation was expressed in %, compared to day 0, which was set at 100%. Production of infectious viral particles was measured in brains (C) and spinal cords (D) every 2 days for 21 dpi. LOD represents the Limit of Detection of infectious viral particles. Results shown are the mean values (with standard deviations) of three independent experiments.

We first sought to investigate the potential biological function of the viral G758R mutation located in the HCoV-OC43 S gene between the S1 and S2 domains, detected in the viral S protein of several clinical isolates from human sputum of upper and lower respiratory tract. Accordingly, we introduced this mutation in the infectious cDNA clone of HCoV-OC43 (pBAC-OC43^FL^) [25] to produce a recombinant mutated rOC/S_G758R_ virus, and we first studied its neuroinvasive and neurovirulent properties compared to reference rOC/ATCC virus (Fig 1). For this, 10 day-old BALB/c mice were inoculated by the intranasal (IN) route [10, 14] and survival curves were obtained (Fig 1A). After infection with the reference virus, over half of BALB/c mice died within the first 15 days post-infection, with symptoms of social isolation and hunched backs. In comparison, the viral mutant was less neurovirulent, with about 30% of mortality. Despite this difference in survival, there were no changes in the symptoms induced by the mutant virus compared to reference strain. Mice were also investigated for variation of weight during infection (Fig 1B) as previously described [14]: mice infected with mutant virus showed a delay in body weight gain of about 50% at 9 days post-infection compared to control mice. Comparison of survival curves of mice infected by both variants, coupled with weight variations, suggested that the rOC/S_G758R_ variant was less neurovirulent than reference virus rOC/ATCC after inoculation by the IN route.

### Viral replication reaches the same level but is delayed for the mutant virus compared to the reference virus after intranasal injection

**Fig 2:**
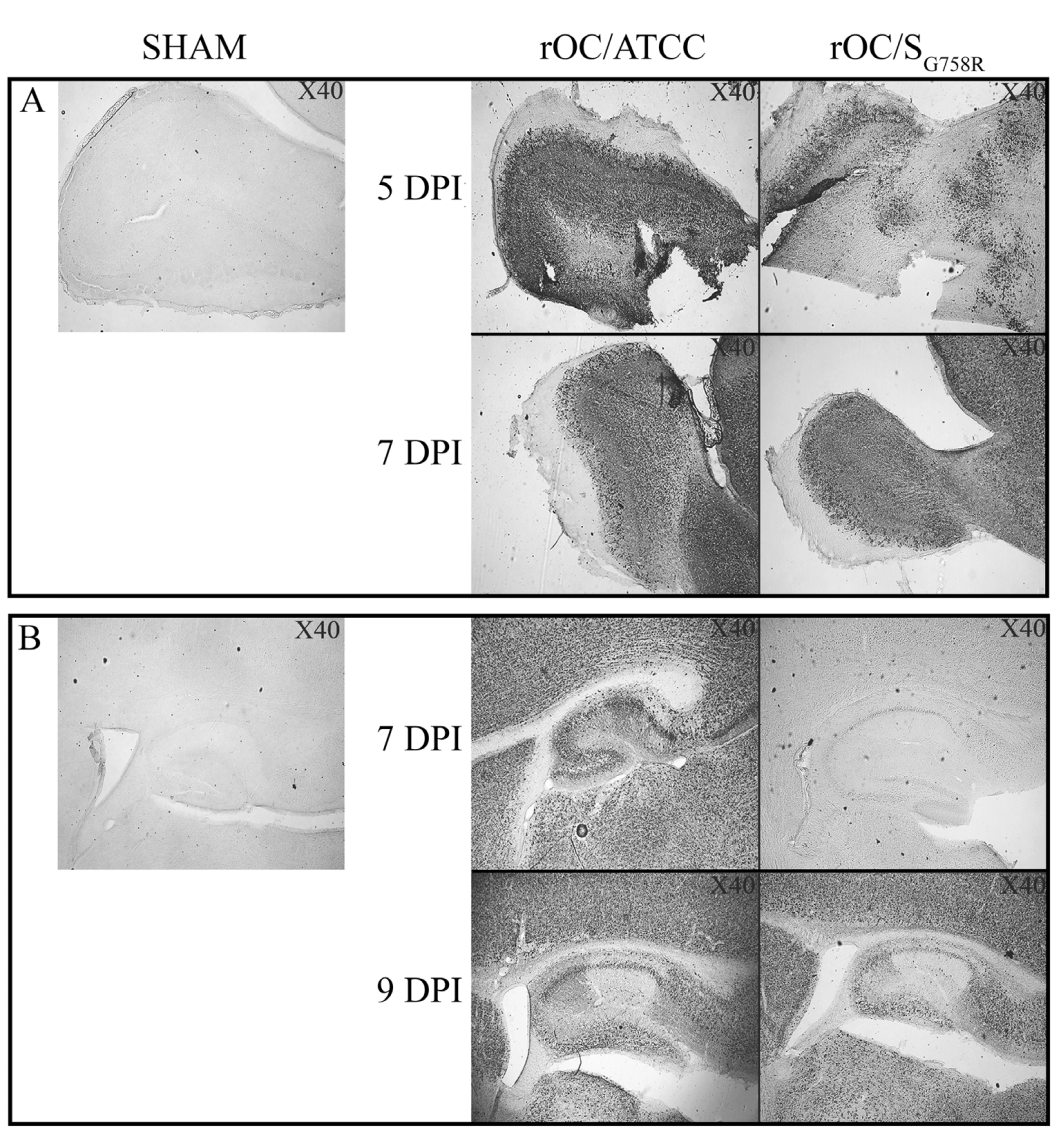
A delay in viral spread is observed in brain of rOC/S_G758R_ -infected mice compared to rOC/ATCC after intranasal inoculation in 10 day-old BALB/c mice. Histological examination of virus spread within the brain of 10-day old BALB/c mice infected with 10^3.25^ TCID_50_/10μL of rOC/ATCC or rOC/S_G758R_, or PBS by the IN route. (A) Detection of viral antigens in the olfactory bulb of infected mice at 5 and 7 dpi. (B) Detection of viral antigens in the hippocampus of infected mice at 7 and 9 dpi. Magnification 40x.

To determine whether the slight difference in neurovirulence between the two viruses could be related to differences in viral replication kinetics within the CNS, brains (Fig 1C) and spinal cords (Fig 1D) were harvested and infectious virus production was evaluated every 2 days over a period of 21 days post-infection (dpi). Even though the difference in neurovirulence between the two viruses did not correlate with a different amount in production of infectious viral particles in the CNS (brain and spinal cord), there was a delay in viral replication kinetics of the mutant virus compared to reference virus. Viral spread in mouse brain (Fig 2) was also studied with a focus on the olfactory bulb and the hippocampus regions, because we have previously determined that these regions are primarily infected by the reference virus strain [14]. At 5 dpi, viral antigens were already present everywhere in the olfactory bulb infected by the reference wild-type virus (Fig 2A), compared to mutant virus for which antigens were only scarcely distributed. At 7 dpi, the kinetics was restored as the mutant infected this region as efficiently as the reference virus. In the hippocampus, we observed the same trend: no viral antigens were detected in this region for the mutant virus at 7 dpi, whereas the spread of both viruses was similar at 9 dpi (Fig 2B). When viruses had spread to all regions of the brain, activation of astrocytes and microglial cells was evident in all infected regions (S1 Fig). Even though no precise quantitation was performed, a slight increase in the number of astrocytes was observed in the olfactory bulb (S1A Fig) and in the hippocampus (S1B Fig) of mice infected by the reference virus compared to mutant virus. Activation of microglial cells was evident in the hippocampus region for both variants at 9 dpi (S1C Fig). As the mutant virus S protein harbors a SNP present in respiratory clinical isolates, we also evaluated viral dissemination towards the respiratory tract. Neither infectious virus particles nor viral RNA were detectable in the lungs of all the mice tested.

### Reference virus rOC/ATCC is more neurovirulent in 21 day-old BALB/c female mice inoculated by the intracerebral route

**Fig 3:**
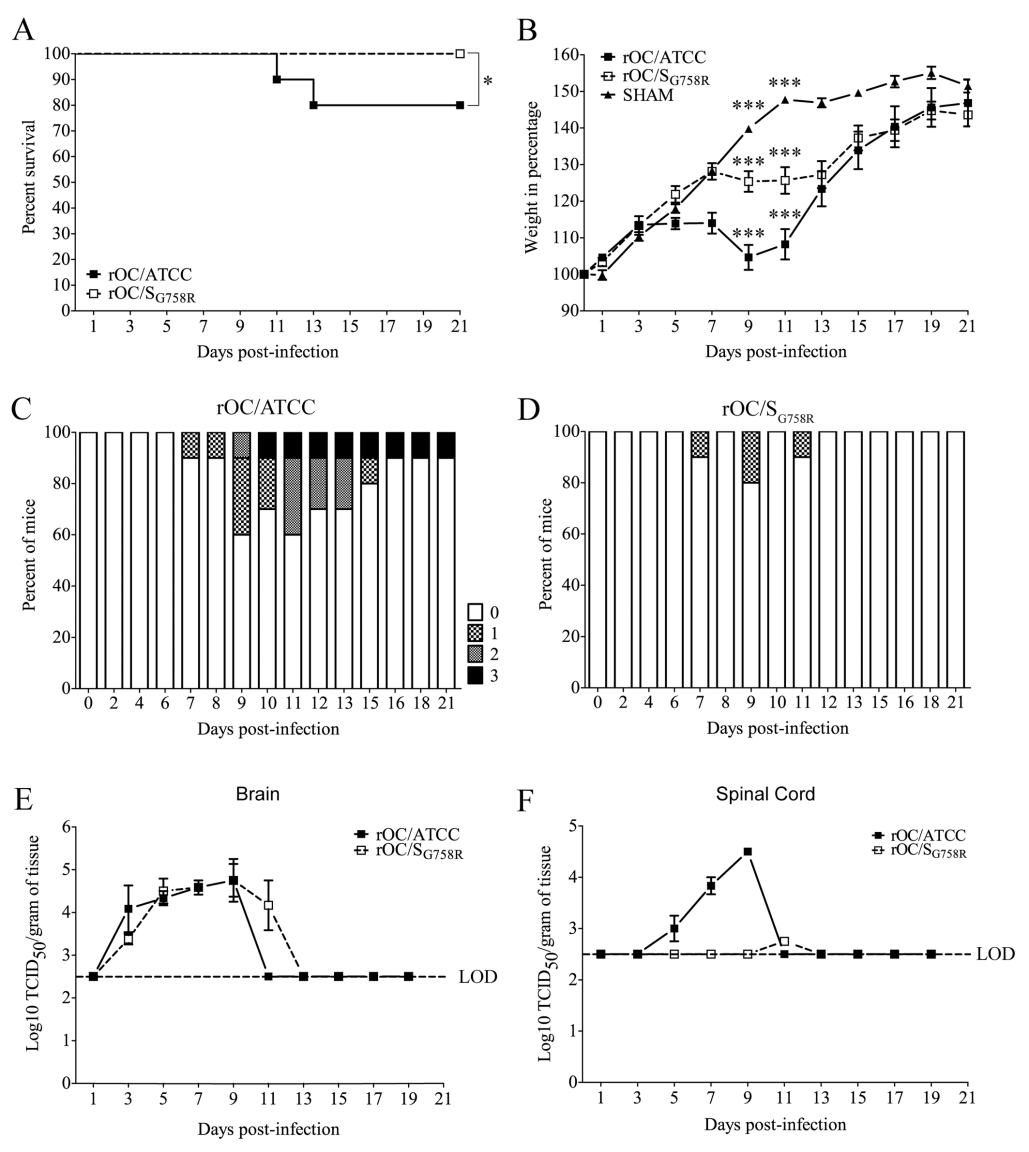
A decreased neurovirulence is observed for rOC/S_G758R_ variant in 21 day-old BALB/c female mice infected by the intracerebral route. 21 day-old BALB/c mice received 10^2.5^ TCID_50_/10μL of rOC/ATCC or rOC/S_G758R_ or PBS by the IC route. (A) Survival curves of mice in % during 21 day post-infection. Difference between the two virus variants was significant (* P≤0.05). (B) BALB/c mice were weighed every 2 days during 21 dpi to estimate weight variations, which were expressed in %, compared to day 0 (100%). Differences were significant (*** P≤0.001) when the three conditions (rOC/ATCC, rOC/S_G758R_ or PBS) were compared between 9 and 11 dpi. Evaluation of the clinical scores (percentage of mice at each level of the scale) of mice infected by rOC/ATCC (C) or rOC/S_G758R_ (D) based on neurological symptoms described in clinical score scale between level 0 and 3 (see Materials and Methods section). Production of infectious viral particles was measured in brains (E) and spinal cords (F) every 2 days for 21 dpi. LOD represents the Limit of Detection of infectious viral particles. Results shown are the mean values (with standard deviations) of three independent experiments.

Having demonstrated that both virus variants retained their neuroinvasive and neurovirulent capacities in our mouse model after intranasal (IN) inoculation, we sought to study the spreading and neurovirulent capacities of the two recombinant viruses after intracerebral (IC) inoculation, as this route results in a more reproducible infection associated with a better control of viral doses introduced into the brain. In order to do so, 21 day-old female BALB/c mice were used [11] and experiments were performed by characterizing mouse survival and weight curves, clinical symptoms of encephalitis and viral replication in brain and spinal cord (Fig 3).

There was a significant difference in survival after inoculation with either virus (Fig 3A): the mutant virus, like the sham control, induced no mortality compared to reference virus, which led to a 20% mortality rate over a period of 21 days. We then measured the weight of mice during the infection (Fig 3B), and observed that there was a significant delay in body weight gain for the reference virus and the mutant virus compared to the sham control between 7 and 11 dpi, which correlates with the survival curves. Using a clinical score scale based on neurological symptoms of mice described in the Materials and Methods section [26], we next studied the clinical symptoms of mice after injection of both variants (Fig 3C and 3D). The only clinical sign caused by mutant virus was the abnormal flexion of the four limbs (level 1) whereas mice infected by the reference virus developed encephalitis associated with the 4 different levels of clinical scores. No clinical signs were noted for sham mice. Taken together, survival and weight curves coupled with the clinical scores indicate that the mutant virus was less neurovirulent than reference virus after IC inoculation in 21-day old mice.

### Viral replication is delayed for the mutant virus compared to the reference virus after intracerebral inoculation in 21 day-old female BALB/c mice

**Fig 4:**
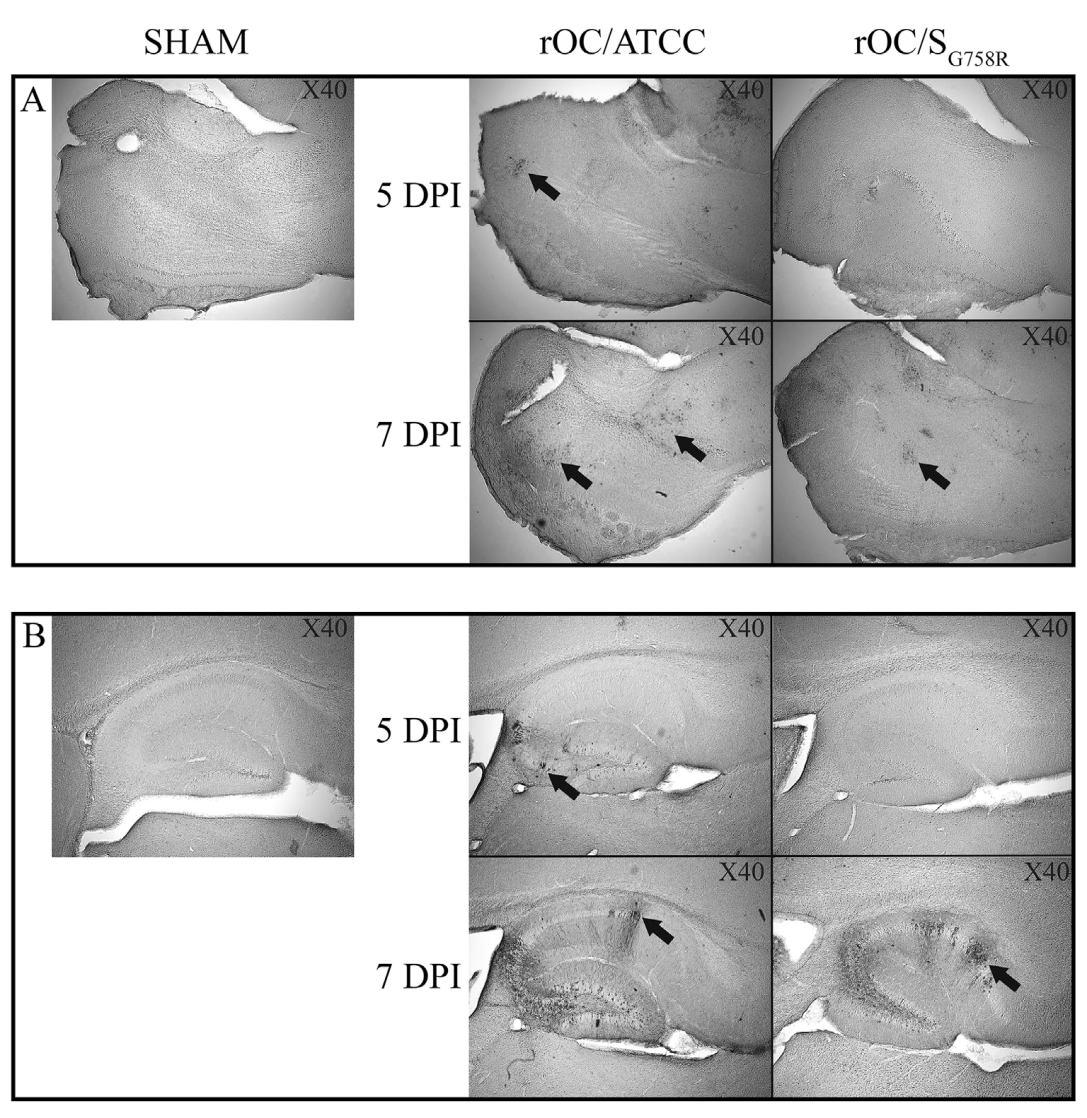
A delay in viral spread is observed in brains of rOC/S_G758R_ -infected mice compared to rOC/ATCC after intracerebral infection in 21 day-old BALB/c female mice. Histological examination of virus spread within the brain. 21 day-old BALB/c mice received 10^2.5^TCID_50_/10μL of rOC/ATCC or rOC/S_G758R_, or PBS by the IC route. Detection of viral antigens in the olfactory bulb (A) or in the hippocampus (B) of infected mice at 5 and 7 dpi at magnitude X40. Black arrows indicate viral particles staining for the S protein.

Given our observation that reference virus was more neurovirulent compared to the mutant virus after inoculation by the IC route, we wished to evaluate whether this correlated with a difference in viral replication in the CNS. Brains and spinal cords were harvested and infectious virus titers were assayed every 2 days for a period of 21 dpi (Fig 3E and 3F). The difference in neurovirulence did not correlate with a significant difference in the amount of infectious viral particles in the brain (Fig 3E). However, there was a drastic difference in the production of infectious virus between both variants in the spinal cord (Fig 3F): virus titers of the reference strain (rOC/ATCC) were almost identical to what was detected in the brain, whereas the less virulent mutant (rOC/S_G758R_) reached the spinal cord only in one out of thirty infected mice. In this mouse, an important delay and a production of viral infectious particles close to the limit of detection suggested that mutant virus had difficulty reaching this portion of the CNS. Histological examination of infected mice revealed that the infected regions were similar following infection by both viruses in the brain, but that the kinetics were different (Fig 4). Indeed, as was the case after the IN route of infection, the IC route of infection also led to a delay in viral replication in the olfactory bulb and in the hippocampus, as no viral antigens were detected before 7 dpi for the mutant virus (compared to 5 dpi for the reference virus). As in 10 day-old BALB/c mice infected IN, when virus had spread to all regions of the brain, activation of astrocytes and microglial cells was evident in all infected regions (S2 Fig). As seen in 10 day-old mice after IN inoculation, even though no precise quantitation was performed, a slight increase in the number of astrocytes in the olfactory bulb (S2A Fig) and in the hippocampus (S2B Fig) could be observed in brains of mice infected by reference virus compared to the mutant virus. The same was observed for microglial cells at 7 dpi in the olfactory bulb (S2C Fig) and in the hippocampus (S2D Fig).

### The predominant G758R mutation in the spike glycoprotein induces an increase in infectious virus release associated with a functional cleavage site

**Fig 5:**
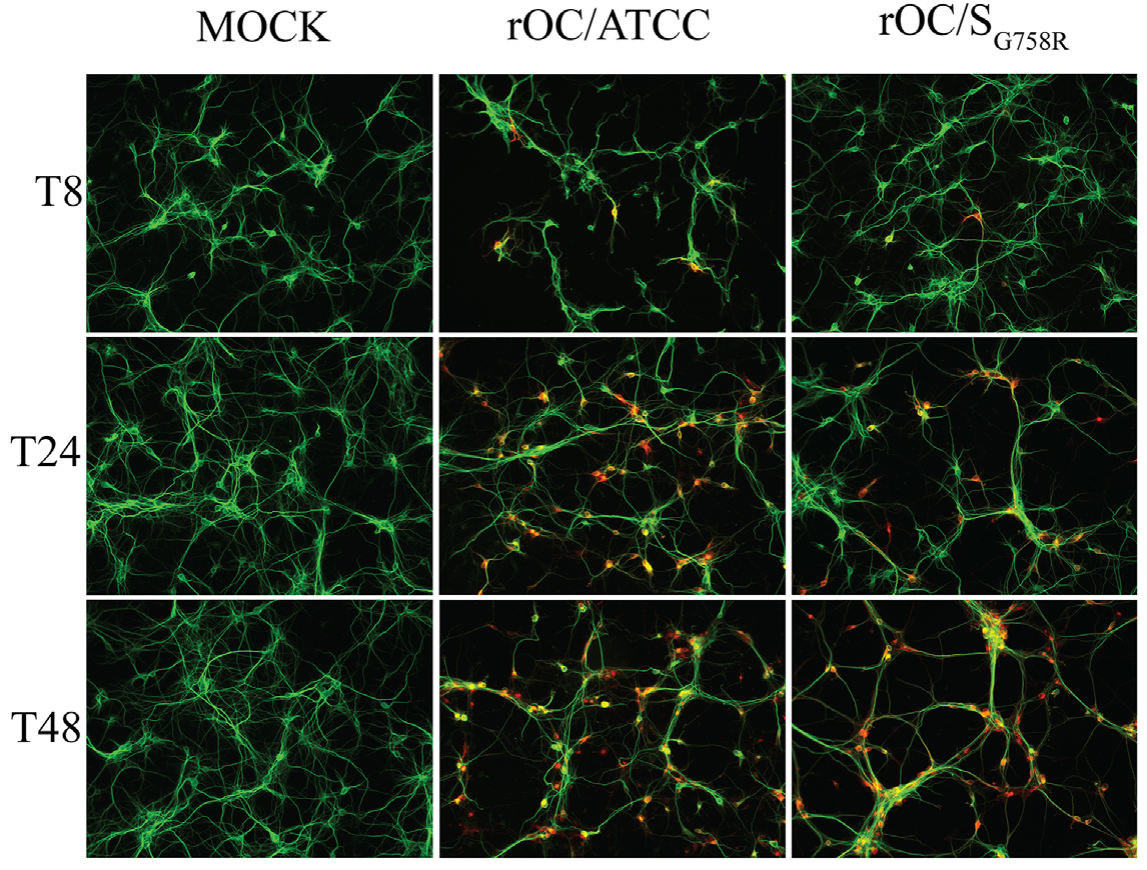
Mutation in the spike glycoprotein of mutant virus delays viral spreading compared to the reference strain in mixed primary CNS cultures from BALB/c mice. Mixed primary cultures from BALB/c mice brain were infected with rOC/ATCC or rOC/S_G758R_ at MOI 0.03. Viral spread was evaluated at 8, 24 and 48 hpi. Neurons were stained in green with a mAb against microtubule-associated protein 2 (MAP2) antibody and the S viral glycoprotein in red, was detected with a rabbit antiserum. Results are representative of three independent experiments. Magnification 200x.

**Fig 6:**
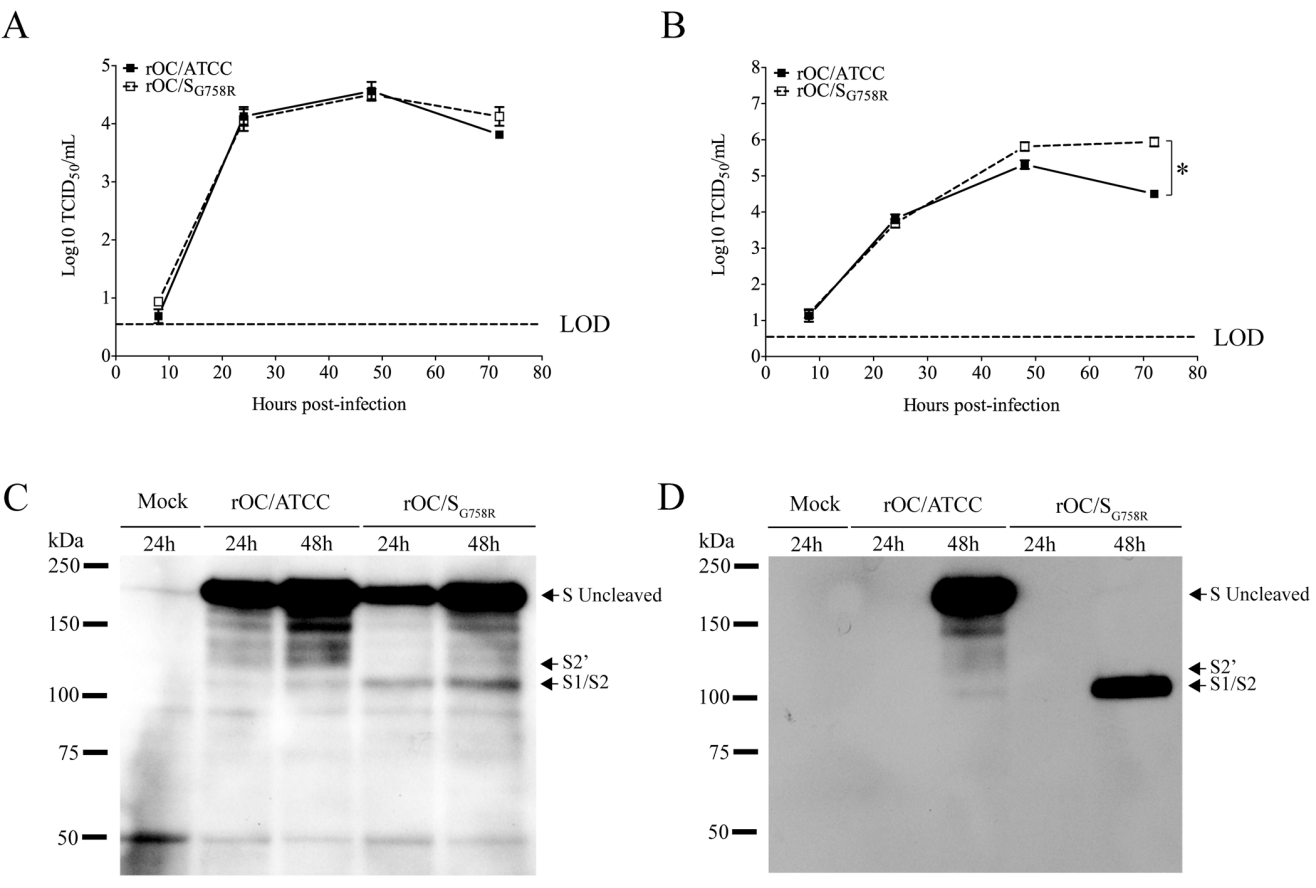
The S glycoprotein harboring a predominant point mutation found in clinical isolates was cleaved more efficiently in supernatant of CNS cells. Mixed primary cultures from BALB/c mice brain were infected with rOC/ATCC or rOC/S_G758R_ at MOI 0.03. Kinetics of infectious virus production in cell-associated (A) and in cell culture supernatant (free virus) (B) was performed. Release of free virus in the supernatant was significantly higher for rOC/S_G758R_ compared to rOC/ATCC (* P≤0.05). (C) Western blot analysis of whole cell lysates (C) or cell culture supernatant (D) (10 μg of proteins) revealed the presence of the uncleaved form of the S glycoprotein (180 kDa), and of a cleaved form at around 100 kDa (S1/S2). Results shown are the mean values (with standard deviations) of three independent experiments.

**Fig 7:**
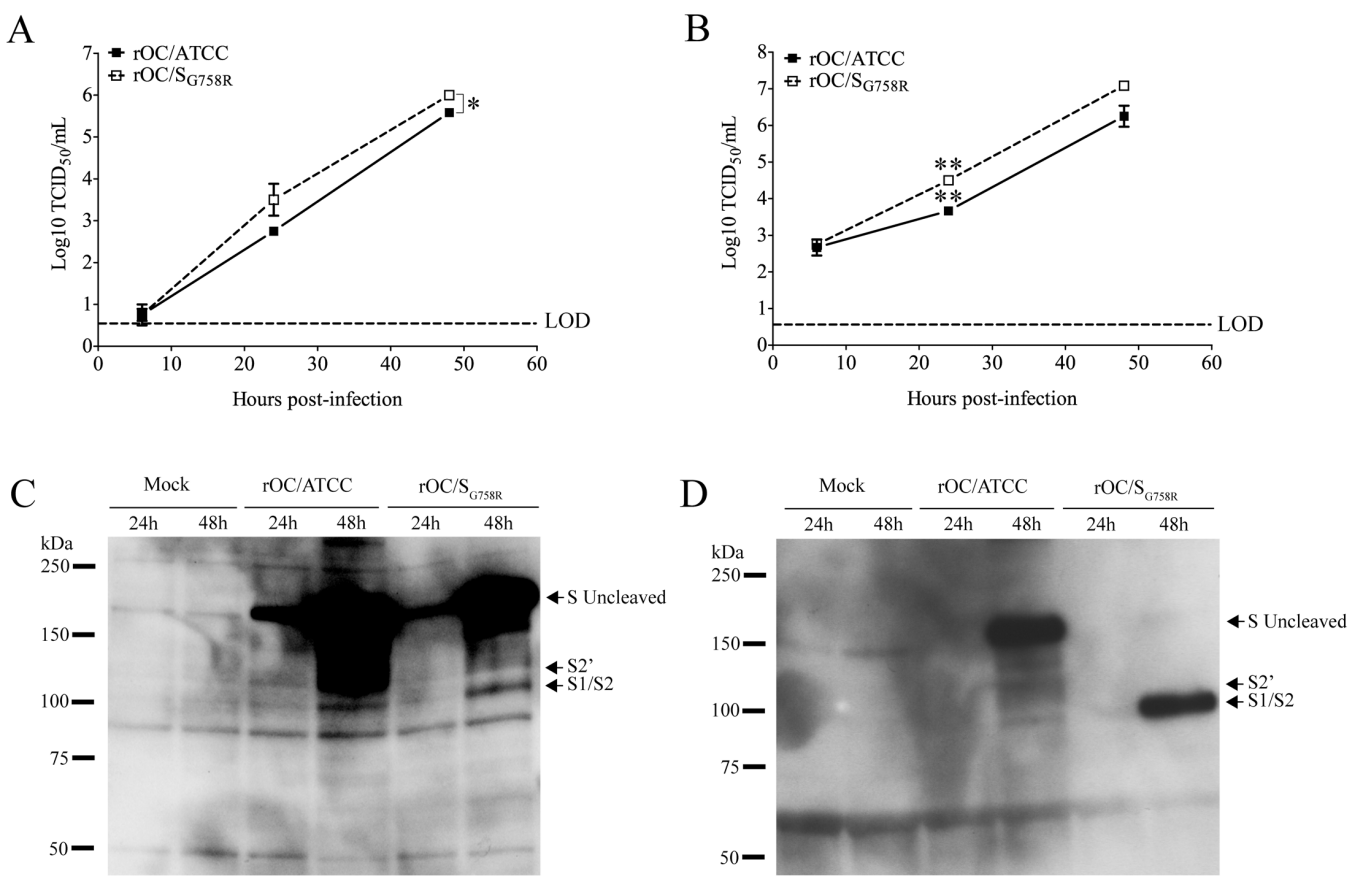
Cleavage of S glycoprotein is also observed in human LA-N-5 cells for mutant virus. The differentiated human neuroblastoma cell line (LA-N-5) was infected with rOC/ATCC or rOC/S_G758R_ at MOI 0.1. Proteins in association with cell or in supernatant were extracted at 24 and 48 hpi, and kinetics of viral replication was evaluated over a period of 48 hpi for (A) cell-associated virus or (B) free virus (supernatant). Titers of cell-associated and free virus were significantly higher for rOC/S_G758R_ compared to rOC/ATCC (* P≤0.05 and ** P≤0.01). Western blot analysis of whole cell lysates (C) or cell culture supernatant (D) (10 μg of proteins) revealed the presence of the uncleaved form of the S glycoprotein (180 kDa), and of a cleaved form at around 100 kDa (S1/S2). Results shown are the mean values (with standard deviations) of three independent experiments.

**Fig 8:**
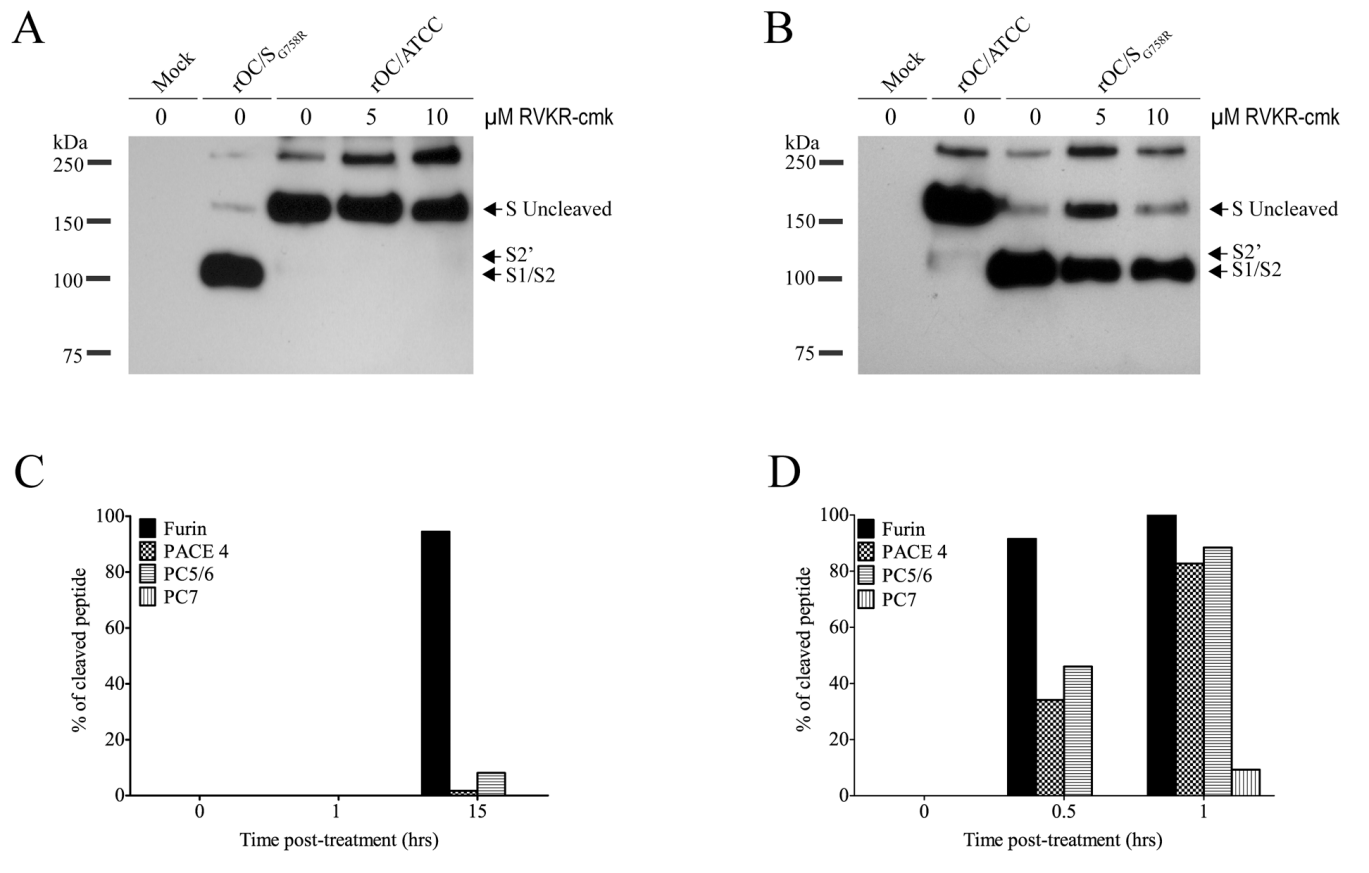
Proprotein convertase as a potential player in S glycoprotein cleavage. (A-B) Differentiated human neuroblastoma cell line (LA-N-5) was incubated before and after infection with different concentration of furin-like inhibitor (dec-RVLR-cmk; 0, 5, 10μM). Infection was performed with rOC/ATCC or rOC/S_G758R_ at MOI 0.1. Proteins in supernatant were extracted at 48 hpi and analyzed by Western blot (50 μg of proteins) of supernatant from LA-N-5 cells infected by reference virus (A) or mutant virus (B). Western blot was directed against viral S glycoprotein. (C-D) Incubation of synthetic peptide containing the sequence of the putative cleavage site of reference virus (C) and mutant virus (D) with different recombinant proprotein convertases *in vitro*. The % of cleaved peptide was quantified over time.

In order to further study the role of the G758R mutation on the biology of both HCoV-OC43 variants, we first evaluated the kinetics of viral replication and spread within mixed primary CNS cultures from BALB/c mice over a period of 72 h post-infection (hpi). Using immunofluorescence, we observed no change in cell tropism, with neurons remaining the main target of infection by both virus variants (Fig 5), even though astrocytes could also be infected later in the infection (S3 Fig) as we previously reported [10]. Interestingly, we did observe a delay in viral spread in neurons for the mutant virus at 8 and 24 hpi compared to the reference strain (Fig 5). Interestingly, even though the infection was shown to be productive for both variants in primary CNS cultures from BALB/c mice, there was a significant increase in the total amount of infectious virus in the cell culture supernatant (free virus) between 48 and 72 hpi for the mutant virus compared to the reference virus rOC/ATCC (Fig 6B). As the G758R mutation creates a putative furin-like cleavage site [16] in the S glycoprotein previously reported to influence viral infectivity [20–22, 24], we wished to evaluate whether cleavage was indeed associated with the delayed spreading in neuronal cells, the increased release of infectious virus and eventually with neurovirulence. As seen in Fig 6, our data correlated with a much stronger cleavage of the S protein of the rOC/S_G758R_ mutant into S1/S2 fragments, compared to reference virus rOC/ATCC at 24 and 48 hpi (Fig 6C; whole cell lysate), which was even more obvious at 48 hpi in the cell supernatant (Fig 6D). In order to evaluate whether this cleavage of the viral S protein also took place in human cells, we made use of the differentiated LA-N-5 neuronal cell line described in the Materials and Methods section [27] and showed, first, that the kinetics of viral replication was similar to that observed between both viruses in murine primary cells (Fig 7A and 7B), as there was a significant increase of virus release for the rOC/S_G758R_ mutant and, second, that the cleavage of the S protein into S1/S2 fragments was again predominantly detected in the cell culture supernatant (Fig 7D) compared to the protein associated with cells (Fig 7C). Again, this cleavage was more evident for mutant than for reference virus. Similar results were obtained with HRT-18 cells. Even though the S protein of HCoV-OC43 reference virus was present mostly in the uncleaved form, our results also show that there are intermediate size bands between the uncleaved and furin-like cleaved forms of the protein. These secondary bands may be unspecific degradation products, but we suggest that among these intermediate size fragments seen on SDS-PAGE, there could be a fragment corresponding to the S protein cleaved at a potential alternative site (S2’ in Figs 6, 7, 8 and S4, the latter showing corresponding overexpositions).

### Proprotein convertases (PCs) are proteases that potentially cleave the HCoV-OC43 S glycoprotein at the putative furin-like cleavage site

In an attempt to determine whether the mutation identified at amino acid 758 (G758R) in the viral S protein could indeed create a furin-like cleavage site, we used a cell-permeable general inhibitor of furin-like PCs to investigate the potential involvement of these proteases in the process. Differentiated LA-N-5 cells were infected in the presence of different concentrations of the decanoylated furin-like inhibitor (dec-RVKR-cmk), and at 48 hpi, proteins in the supernatant were harvested and analyzed (Fig 8). As expected, the S glycoprotein of the reference virus was not cleaved at all (Fig 8A) whereas the cleavage of the S protein of mutant virus was inhibited in a dose-dependent manner by dec-RVKR-cmk (Fig 8B). To evaluate whether the kinetics of viral replication was also affected, supernatants were harvested over a period of 48 hpi, and evaluation of infectious viral particles revealed no significant differences (S5 Fig). As the furin-like inhibitor reduced the cleavage of the S protein for the rOC/S_G758R_ variant, we sought to identify which proprotein convertase(s) could play a role in cleavage of the S glycoprotein during infection. As shown in Fig 8C and 8D, a synthetic peptide containing the sequence of reference virus (RRSRG), was only cleaved by recombinant furin after 15 hours, likely at RRSR↓G. On the other hand, the synthetic peptide containing the sequence of mutant virus (RRSRR) was cleaved in only 30–60 minutes, likely at RRSR↓R, by furin and less so by three additional proprotein convertases: PACE4, PC5/6 and much less efficiently by PC7.

### Differential cleavage of the S glycoprotein leads to a modification in morphology of the viral particle

**Fig 9:**
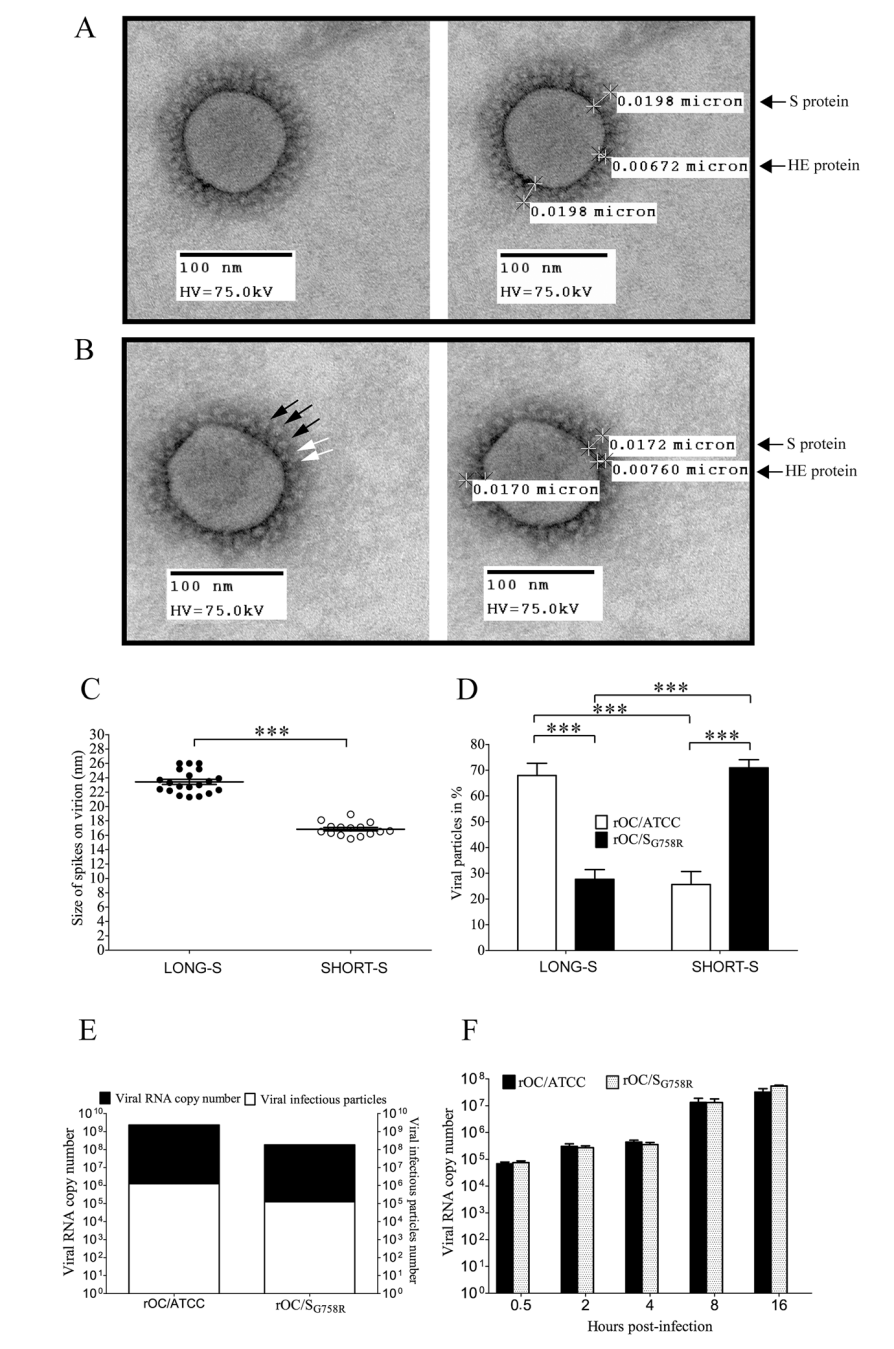
Morphology of viral crown peplomers is dependent on cleavage of the S glycoprotein but does not affect virus infectivity. Observation of virus crown peplomers was made by Transmission Electron Microscopy (TEM). Mixed primary cultures from BALB/c mice brain were infected with rOC/ATCC or rOC/S_G758R_ at MOI 0.03 and supernatants were harvested at 48 hpi. After negative staining by PTA, viral particles were observed. (A) Left panel represents the “long S” morphology at magnification 50,000x and right panel indicates spike size in μm on the same viral particle. (B) Left panel shows the “short S” morphology at magnification 50,000x and right panel indicates spike size in μm on the same viral particle. Black arrows represent different sizes of spike glycoprotein, and white arrows show the viral hemagglutinin-esterase (HE) protein peplomers. (C) Mean size of spike glycoprotein (in nm) was evaluated for long S and short S types of viral particles. Measurements between 20 and 26 nm in length were considered as long S and those between 14 nm and 20 nm in length were considered as short S spikes on viral particles. The difference between long S and short S viral particles was significant (*** P≤0.001). (D) Quantification of 650 long S and shortS viral particles of rOC/ATCC or rOC/S_G758R_ shows a significant difference between both viruses (*** P≤0.001). (E) Infectivity assay between viruses: quantification of viral RNA (absolute quantity in RNA copy) over the number of infectious particles in viral stocks. (F) The amount of viral RNA copy found associated with LA-N-5 cells during the early steps of infection at 0.5, 2, 4, 8, 16 hpi. Results are shown in absolute number of viral RNA copy.

Having shown that proprotein convertases are able to cleave the viral S glycoprotein *in vitro*, we sought to determine whether this cleavage could be associated with a change in viral particle morphology for the rOC/S_G758R_ variant compared to reference rOC/ATCC. Our observations by transmission electron microscopy (TEM), suggest that the typical coronavirus double crown-shape of the HCoV-OC43 virion was present in two different forms in cell supernatants that were harvested at 48 hpi during infection of mixed primary CNS cultures from BALB/c mice. Indeed, Fig 9A (left panel) represents the first type of morphology, which we named “long” for long S peplomers as measurements of the spike (S) and the hemagglutinin-esterase (HE) peplomer is shown for the same particle in the right panel. The same relative length of S and HE proteins were previously determined for other coronaviruses [28]. The second type of crown morphology, which we named “short” for short S peplomers is presented in Fig 9B. This short S morphology shows normal HE peplomers of similar length. For a more accurate characterization of the spike length on viral particles from both variants, we measured the spike length of an equal number of virions (10 for each virus), for which the crown presented long (long S) or short (short S) peplomers (Fig 9C). The average length of the spike associated with a long S type of crown morphology was 24 nm, whereas the average length of the spikes on short S virions was about 15 nm. This apparent average difference of 9 nm represents a reduction of about 37.5% in the total length of the spike, which could presumably play a role in viral infectivity. Therefore, we next counted the number of viral particles that have a long-S or short-S crown for both viruses (Fig 9D) and found a significant difference, which tended to demonstrate that viral particles of the mutant virus were mostly in the short S state (about 72%) compared to the reference virus, which showed mainly long S crown (about 68%).

Given that the rOC/S_G758R_ variant was less neurovirulent and presented a delay in dissemination within the mouse CNS compared to rOC/ATCC reference virus, two observations that can relate to a difference in viral infectivity in neuronal cells, we sought to evaluate whether there was a correlation between the morphological differences of the crown of viral particles (Fig 9A–9D) and their relative infectivity (capacity to infect the target cell). No significant differences were found in the ratio of infectious viral particles over total viral particles (evaluated by the number of viral genome present in viral stocks used for all experiments), which establishes itself at 1/200 for both variants (Fig 9E). Furthermore the amount of viral RNA associated with infected LA-N-5 cells remained the same over a period of 16 hours (Fig 9F), suggesting that attachment and cell entry was similar for both viruses.

## Discussion

Being opportunistic pathogens, HCoV are naturally neuroinvasive and neurotropic in humans [5]. Herein, making use of our cDNA infectious clone, we show that a single nucleotide polymorphism (SNP) naturally found in the S gene of all known HCoV-OC43 contemporary clinical isolates leads to a G758R mutation in the S protein, without significantly affecting the virus neuroinvasive properties and infectivity in cell culture. However, this mutation was sufficient to modify viral spread and neurovirulence in susceptible mice by modulating the cleavage of the S protein, which appears related to furin-like activity in susceptible neuronal cells.

Even though the rOC/S_G758R_ mutant harbors a SNP present in respiratory clinical isolates, we were not able to detect any viral presence in the respiratory tract of infected mice. Further studies are underway to try to identify other naturally occurring S mutations that could be important for viral spread to the respiratory tract in mice. Nevertheless, our results indicate that, despite the difference in neurovirulence, the recombinant virus rOC/S_G758R_ retains its full neuroinvasive properties even though there was a delay in viral spread and in the production of infectious virus (Figs 1–4). This phenomenon may in part explain the mutant reduced neurovirulence accompanied by less severe neurological symptoms and a less frequent spread to the spinal cord, as previously reported for other S protein mutants of HCoV-OC43 [14] and for the murine coronavirus, MHV [29].

When viruses had spread to all regions of the brain, the innate immune response was well established, as observed by astrogliosis and microgliosis after both routes of infection where we detected viral antigens [10, 14, 30] and S1 and S2 Figs. The stronger astrogliosis and microgliosis observed after infection by the reference virus may also be related to a faster spread throughout the CNS compared to the mutant virus [14]. The same difference in viral spread was confirmed in primary cultures of mouse brain cells, where both variants were still infecting neurons as primary targets (Fig 5), even though astrocytes could also be infected (S3 Fig). This is in agreement with our previous reports in these cultures [14] and underlines the fact that the change in neurovirulence was not associated with a change in cell tropism as was previously shown for MHV [31], but could rather be related to a modification in the spread between infected neurons. The differential neurovirulence and spread could certainly be the consequence of the G758R mutation in the S protein, which introduces a typical furin-like recognition site [16, 32] probably recognized by cellular proprotein convertases (PCs) as this mutation was the only difference found in the whole genome of both recombinant viruses used in the present study. Proteolytic cleavage of coronaviruses S proteins was characterized several years ago for the murine coronavirus [33]. Since then, several reports have indicated that PCs appear to be important for cell-cell fusion and/or virus entry into host cells [21–23], or during transport of the newly assembled virions through the secretory pathway of the producer cell [21, 34–36] for different coronaviruses including MHV, SARS-CoV and FIPV[21, 23, 37, 38].

The data presented in Figs 6 and 7 clearly show that the S protein harboring the G758R mutation is more easily cleaved during infection. This S1/S2 cleaved version of the S protein is easily detected in the free virus present in cell culture supernatant but it is barely detectable in cell-associated proteins. Taken together, these results strongly suggest that this cleavage of S takes place during the late steps of infection, probably during particle assembly and egress, as it was previously shown for MERS-CoV [35] and MHV [39]. Furthermore, cleavage of the HCoV-OC43 S glycoprotein also has an impact on pathology, as it decreases neurovirulence and spread within the CNS. It is highly interesting to note that this association between decreased virulence and cleavage of coronavirus S glycoprotein was only suggested for FCoV [32]. In fact, for other coronaviruses, including the murine (MHV) and the bovine coronavirus (BCoV), no clear association was established between S cleavage and virulence [40, 41].

The data presented in Fig 8A and 8B strongly suggest that PCs can indeed be involved in the cleavage of HCoV-OC43 S protein during infection of neuronal cells. Inhibition of furin-like protease (PCs) was already demonstrated for other coronaviruses like MERS-CoV and MHV with the same type of inhibitor [21, 35]. These results are supported by observation of S protein in reference virus rOC/ATCC (Fig 8A) for which the ratio of uncleaved S protein over S1/S2 cleaved form remained equal at all inhibitor concentrations. In contrast, this ratio increased for mutant virus rOC/S_G758R_ (Fig 8B) in a dose-dependent manner. Results presented in Fig 8C and 8D bring even more interesting new information about which of these proteases could be involved in the actual cleavage. Indeed, the synthetic peptide harboring the G758R mutation was cleaved with much more efficiency by PCs than the model peptide mimicking the reference virus S protein. Furthermore, even though furin represented the most efficient convertase, PC5/6 and PACE4, and to a much lesser extent PC7, were also able to cleave the synthetic peptide and could therefore cleave the HCoV-OC43 S protein during infection of susceptible cells as it was previously shown for SARS-CoV [42]. Inhibition of furin-like activity during infection of human neuronal cells, lead us to suggest that PCs (most notably furin) are the cognate proteases involved in the S protein cleavage at amino acid 758 (Fig 8).

The number and morphology of glycoproteins on virions can modulate infectivity for different RNA viruses harboring a class I fusion protein, including other coronaviruses [43–46]. In the case of HCoV-OC43, the apparent modification of crown-shaped virions (Fig 9A–9D), associated with the observed differential S protein cleavage, does not seem to increase or decrease viral infectivity (Fig 9E and 9F). Moreover, inhibition of furin-like-activity did not influence the capacity of the viruses to enter these cells (S5 Fig). Therefore, even though the S cleavage associated with furin-like activity was shown to influence viral entry for IBV [24], the HCoV-OC43 S protein cleavage by PCs did not appear to modulate infectivity as it was shown for MERS-CoV [47].

On the other hand, the modified virion morphology associated with preferential cleavage of the rOC/S_G758R_ HCoV-OC43 variant S protein at the S1/S2 domain interface correlates with a decrease CNS viral spread and neurovirulence in susceptible mice. Indeed, the delays in spreading in both primary cultures (Fig 5) and within the CNS (Fig 2) were observed despite a more efficient release of infectious rOC/S_G758R_ particles in the cell culture medium as compared to the reference rOC/ATCC virus, a relationship that may seem counterintuitive but is in fact reminiscent of the cell-to-cell mode of propagation prevailing for a growing list of viruses [48]. For example, HTLV-1 is famously inefficient at spreading through free-virus particles diffusion, the particles remaining instead associated to the plasma membrane from where productive transfer towards target cell occurs [49, 50]. By analogy, it is tempting to speculate that S1/S2 HCoV-OC43 spike cleavage limits the amount of particles at the plasma membrane available for a cell-to-cell transfer to naive neurons. This can explain the difference in kinetics of dissemination between both viruses and the difficulty for mutant rOC/S_G758R_ to reach the spinal cord even though it does disseminate throughout the brain. This hypothesis may appear in contradiction with the previously documented positive impact of S1/S2 spike cleavage on MHV and SARS-CoV cell-to-cell transfer occurring upon fusion-dependent syncytium formation [21, 22, 39]. Furthermore, even though syncytium formation upon MHV infection has often been linked to S cleavage, in some instances, this type of cell-cell fusion was shown to occur without cleavage of the S protein [51–53] and the MHV-2 strain S protein can be cleaved without being able to induce syncytia [54]. In fact, regardless of the cleavage status of its S protein, HCoV-OC43 was never able to induce syncytia in any type of cells we studied and we therefore tend to think that distinct, but not mutually exclusive, cell-to-cell propagation mechanisms may prevail among coronaviruses like it does for other enveloped viruses, especially within the CNS, where cell-cell movement of viruses may take place at synapses [48]. Altogether, these observations suggest that the influence of spike cleavage on coronavirus propagation is not an absolute prerequisite and therefore, cannot *per se* predict accurately the efficiency of cell-to-cell spread. The reasons underlying this variable outcome are still unclear but may well reside in the different virus receptors, structural features and attachment factors exploited by coronaviruses. Given the expected influence of virus spread on neurovirulence, host survival and potentially establishment of CNS viral persistence, further studies are indeed warranted to characterize the underlying mechanisms associated with HCoV-OC43 spread within the CNS.

The SDS-PAGE (Figs 6, 7, 8 and S4) shows intermediate size fragments migrating between the uncleaved and furin-like cleaved forms of the S protein that may represent unspecific degradation products. However, analysis of the S protein gene sequences of HCoV-OC43, revealed a second putative cleavage site (S2’) between amino acid 899 and 903 (KASSR). If functional, this second putative cleavage site could be used by other types of cell proteases, including trypsin, TMPRSS and cathepsins [20, 55–57] to produce a fragment of such molecular weight. Further studies to characterize the possible involvement of this second putative cleavage site and the identification of host proteases involved in the potential processing of the HCoV-OC43 S protein are ongoing.

Taken together, the results of the current study indicate for the first time that HCoV-OC43 is clearly able to infect neuronal cells and to spread with or without the need for a furin-like S protein cleavage. The difference in viral spread within CNS and in brain primary cultures, associated with the increase of infectious viral particles in the culture medium for the virus harboring the G758R mutation present in all known clinical isolates, as well as the absence of modification in infectivity between the two viruses, strongly suggests that the PC-activity-associated cleavage of HCoV-OC43 S protein plays a more important role during the egress and viral budding from infected cells, which could influence the mode of viral transmission between CNS cells. This is of importance to better understand the mechanisms underlying viral spread within the CNS, potentially associated with an adaptation of HCoV-OC43 to this particular environment. Even though HCoV-OC43 reference strain is highly neurovirulent, we have already shown that its RNA persists in the mouse CNS for up to one year in a significant proportion of infected mice [10]. Nevertheless, the delayed dissemination and reduced neurovirulence of mutant rOC/S_G758R_ increase host survival and therefore could favor the establishment of CNS viral persistence associated with a potential viral adaptation to the CNS environment, which could result in the selection of better adapted quasi-species, as it was shown for MHV [58]. In the end, such a persistent infection in the human CNS could, in certain circumstances, be associated with recurrent human encephalitis or neurological degenerative pathologies. Therefore, the observation that HCoVs are naturally neuroinvasive in both mice and humans [9, 59, 60] underlines the need to further characterize viral and cellular determinants of these neuroinvasive properties. Understanding mechanisms and consequences of virus interactions with the nervous system is essential to better understand potentially pathologically relevant consequences and in the design of diagnostic and therapeutic strategies, including modulation of host proteases such as proprotein convertases.

## Materials and Methods

### Ethics statement

All animal experiments were approved by the Institutional Animal Care and Use Ethics Committee (IACUC) of the *Institut National de la Recherche Scientifique* (INRS) and conform to the Canadian Council on Animal Care (CCAC). Animal care and used protocols numbers 1304–02 and 1205–03 were issued by the IACUC of INRS for the animal experiments described herein.

### Viruses and cell lines

The wild-type reference virus HCoV-OC43 (VR-759) was obtained in the 1980s from the American Type Culture Collection (ATCC). The recombinant HCoV-OC43 virus (rOC/ATCC) was generated using the full-length cDNA clone pBAC-OC43^FL^ and displayed the same phenotypic properties as the wild-type virus, as previously described [25]. This recombinant virus was used as the reference control virus for all experiments. Using site-directed mutagenesis (Stratagene QuikChange Multisite-directed mutagenesis kit) as recommended by the supplier, we introduced a point mutation in the gene coding for the spike glycoprotein of HCoV-OC43 at nucleotide 2272, corresponding to an amino acid change at position 758 (corresponding recombinant virus designated rOC/S_G758R_). Each cDNA clone was transfected in BHK-21 cells, amplified by two passages in the HRT-18 cell line, and sequenced to make sure that only the introduced G758R mutation was present and that no other mutations appeared. The HRT-18 cell line (a gift from the late David Brian, University of Tennessee) was cultured in minimal essential medium alpha (MEM-alpha; Life Technologies) supplemented with 10% (vol/vol) fetal bovine serum (FBS; PAA GE Healthcare) and was used to produce viral stocks. The LA-N-5 cell line (a kind gift of Stephan Ladisch, George Washington University School of Medicine) was cultured in RPMI medium supplemented with 15% (vol/vol) fetal bovine serum (FBS), 10 mM HEPES, 1 mM sodium pyruvate, and 100 μM non-essential amino acids (Gibco- Invitrogen). LA-N-5 cells were differentiated into human neurons as previously described [61]. Briefly, cells were seeded in Cell+ petri dishes (5x10^5^ cells in RPMI medium supplemented with 10% (vol/vol) FBS, 10 mM HEPES, 1 mM sodium pyruvate, and 100 μM non-essential amino acids. The next day and every 2 days for 6 days, the medium was replaced with the same medium supplemented with 10% (vol/vol) FBS and 10 μM all-trans retinoic acid (Sigma-Aldrich).

### Treatment of cells with protease inhibitors

Before infection, differentiated LA-N-5 cells in Petri dishes, and 24-well plates were pretreated with furin inhibitor Decanoyl-Arg-Val-Lys-Arg-chloromethylketone (Dec-RVLR-cmk; Bachem N-1505) at different concentrations (5-10-20-40 μM) for 2 h at 37°C. The medium was removed and cells were infected at a defined multiplicity of infection (MOI) of 0.1, with reference and mutant virus and incubated for 2 h at 37°C without furin inhibitor, washed with PBS and incubated at 37°C with fresh RPMI containing Dec-RVLR-cmk at the concentrations used before infection. At 6, 24 and 48 hpi, supernatants and cells were harvested separately for protein extraction and evaluation of infectious virus production.

### Mixed primary cultures of mouse CNS cells

Embryos at 14 to 16 days of gestation were removed from pregnant anesthetized CD1 mice. The cortex and hippocampus of the embryonic pup brains were harvested and placed in Hanks balanced salt solution (HBSS) medium, without Ca^2+^ and Mg^2+^, supplemented with 1.0 mM sodium pyruvate and 10 mM HEPES buffer. The tissues were incubated in 5 ml of HBSS+trypsin-EDTA 0.5% (ratio 10:1 respectively) for 15 min at 37°C with gentle tilting to mix. After digestion, the tissues were washed 5 minutes three times with HBSS, and the medium was removed and replaced by fresh HBSS medium (without Ca^2+^ and Mg^2+^, supplemented with 1.0 mM sodium pyruvate and 10 mM HEPES buffer). Tissues were gently pipetted up and down with a Pasteur pipette to dissociate the cells. After a decantation step of 5 min at room temperature, supernatants were transferred in a 50 ml tube with 36 mL of neurobasal medium (Invitrogen) supplemented with 0.5 mM GlutaMAX-I (Life Technologies), 10 mM HEPES buffer, B27 supplement (Life Technologies), gentamycin and 10% (vol/vol) of Horse serum (Life Technologies). This step was performed twice to increase the final amount of cells. Cells were then seeded at 2x10^5^ cells/cm^2^ and grown on collagen+poly-D-lysine (3:1 for a final concentration at 50 μg/mL for both)-treated glass coverslips in the same medium, which was replaced by fresh neurobasal medium without horse medium the next day. The medium was changed every 2 days after and the cultures were ready for infection after 7 days in culture.

### Infection of human cell lines and primary mouse CNS cultures

The HRT-18 and LA-N-5 cells as well a primary mouse CNS cell cultures were infected at a defined MOI of 0.1, or mock-infected and then incubated at 33°C (HRT-18) or 37°C (LA-N-5 cell line and primary cultures), for 2 h (for virus adsorption), and incubated at 33°C with fresh MEM-alpha supplemented with 1% (vol/vol) FBS (for HRT-18 cells) or at 37°C with fresh neurobasal medium with B27-GlutaMAX-I (for primary murine CNS cell cultures) or at 37°C with fresh RPMI medium supplemented with 2.5% (vol/vol) FBS (for LA-N-5 cells) for different periods of time.

### Mice, survival curves, body weight variations and evaluation of clinical scores

Female BALB/c mice (Jackson Laboratories) aged 22 days post-natal (dpn) or 10 dpn were inoculated respectively by the IC route with 10^2.5^ or the intranasal route with 10^3.25^ of 50% tissue culture infective doses (TCID_50_) recombinant virus, as previously described [14]. Groups of 10 mice infected by each recombinant virus were observed on a daily basis over a period of 21 dpi, and survival and weight variations were evaluated. Clinical scores were evaluated using a scale with 4 distinctive levels (0 to 3); where 0 was equivalent to the asymptomatic mouse; 1 for mice symptoms of abnormal flexion of the four limbs [10]. Mice presenting social isolation, ruffled fur, hunched backs and weight loss were classified as number 2 and number 3 was attributed to mice that were in moribund state or dead. This neurological scale was adapted from Burrer *et al*. already published for several viruses [26].

### Evaluation of infectious virus production

Mouse brain and spinal cord tissues or cell culture supernatants were processed for the presence and quantification of infectious virus by an indirect immunoperoxidase assay (IPA) on HRT-18 cells, as previously described [62]. Briefly, HRT-18 cells were incubated with the mouse primary antibody 4.3E4 (dilution 1/50) that detects the S protein of HCoV-OC43. After three PBS washes, cells were incubated with a secondary horseradish peroxidase-conjugated goat anti-mouse immunoglobulin antibody diluted 1/500 (Kirkegaard & Perry Laboratories). Finally, immune complexes were detected by incubation with 0.025% (wt/vol) 3,3-diaminobenzidine tetrahydrochloride (Sigma-Aldrich) and 0.01% (vol/vol) hydrogen peroxide in PBS 1X, and infectious virus titers were calculated by the Karber method, as previously described [62].

### Immunohistochemistry/immunofluorescence

For immunohistochemistry, perfusion with 4% (wt/vol) paraformaldehyde (PFA) was performed on five infected BALB/c mice for each recombinant virus, every 2 days, between 1 and 15 dpi. Sagittal brain sections were prepared at a thickness of 60 μm with a Lancer Vibratome. Serial sections were collected and incubated overnight with primary antibodies, as previously described [11]. For detection of viral antigens, 1/1000 dilutions of ascites fluid from the 4.E.11.3 hybridoma secreting a murine monoclonal antibody against the viral N protein were used [8]. Astrocytes were identified with a rabbit anti-glial fibrillary acidic protein antibody (GFAP; Dako) diluted 1/500, and activated macrophages/microglia by a rabbit anti-Iba 1 (Wako) diluted 1/500 for 10 day-old BALB/c mice, or a rat anti-Mac 2 monoclonal antibody diluted 1/50 for 21 day-old female BALB/c mice. For immunofluorescence staining, primary murine CNS cell cultures were washed with sterile PBS and then fixed with 4% (wt/vol) paraformaldehyde for 30 min at room temperature. After washing, cells were permeabilized with 100% methanol at -20°C for 5 min. The samples were then incubated with primary antibodies: a polyclonal rabbit anti-glial fibrillary acidic protein (GFAP) antibody (1/1000; Dako), and a monoclonal mouse anti-S protein antibody (1/2 of 4.3.E4 hybridoma supernatant) (S3 Fig) or polyclonal rabbit anti-S protein of the bovine coronavirus (BCoV) at 1/1000 dilution and a monoclonal mouse anti-microtubule-associated protein 2 (MAP2) antibody (at a dilution of 1/1000), 1 h at room temperature. After three washes with PBS, cells were incubated in the dark for 1 h at room temperature with the secondary fluorescent antibodies Alexa Fluor 568 goat anti-rabbit (1/1000; Life Technologies) or Alexa Fluor 488 anti-mouse (1/1000; Life Technologies). After three PBS washes, tissue sections were incubated for 5 min at room temperature with 4’,6-diamidino-2 phenylindole (DAPI; 1 μg/ml; Life Technologies) washed once with PBS and water and then mounted with Immuno-Mount mounting medium (Fisher Scientific). Immuno-histochemical and fluorescent staining were observed under a Nikon Eclipse E800 microscope with a QImaging Retiga-EXi Fast 1394 digital camera using Procapture system software.

### Protein extraction and western blot analysis

Proteins in the cell culture medium and cell-associated proteins were extracted using RIPA buffer (150 mM NaCl, 50 mM Tris, pH 7.4, 1% (v/v) NP-40, 0.25% (w/v) sodium deoxycholate, 1 mM EDTA) supplemented with the Protease cocktail inhibitor (Sigma) and the Halt-Phosphatase inhibitor (Pierce). Harvested cells were pipetted up and down into RIPA buffer, incubated on ice for 20 min, centrifuged for 10 min at 4°C at 17,000×g and supernatants were stored at −80°C until further analyzed. Protein concentrations were determined using the BCA Protein assay kit (Novagen), according to the manufacturer's instructions. Equal amounts of proteins were subjected to SDS-PAGE using a Criterion 4–12% gradient gel, or a Tris-Glycine 4–15% gradient gel, transferred to PVDF membrane with a semi-dry trans-blot apparatus (BIO-RAD). Membranes were blocked overnight at 4°C with TBS buffer containing 1% (v/v) Tween (TBS-T) and 5% (w/v) non-fat milk, then incubated with the monoclonal mouse anti-S protein antibody 4.3E4 (hybridoma supernatant 1/2) for 1 h at room temperature. After three washes of 10 min with TBS-T, the membranes were incubated with a secondary anti-mouse antibody coupled to horseradish peroxidase (GE Life Sciences) and detection was performed using the enhanced chemiluminescence (ECL) kit (BIO-RAD) using Kodak-X-Omat L-S film (Kodak).

### Transmission electron microscopy

For the observation of viral particles by Transmission Electron Microscopy (TEM), 200 μL of the supernatant of infected mixed primary cultures of murine CNS were ultracentrifuged on a nickel grid at 50,000 rpm for 5 min. The grids were then dried with bibulous paper before negative staining of 1 min with a drop of 3% phosphotungstic acid (PTA).

### Infectivity assay

Real time RT-PCR for the absolute quantitation of viral RNA (genome) in viral stocks and during infection of LA-N-5 cells, was modified from Vijgen and collaborators [63] using the Taqman technology and the use of cRNA standards for the generation of a standard curve and to evaluate the absolute number of viral genome in samples with the MEGAshortscript kit (Ambion/Life Technologies) [63, 64]. Briefly, total RNA was extracted with the Qiazol reagent (Qiagen) for HRT-18 cell culture supernatant to evaluate the amount of viral genome in virus stock and with Qiagen RNeasy mini extraction kit according to the manufacturer's instructions for total RNA extraction following infection of LA-N-5 cells at 0.5, 2, 4, 8, and 16 hpi. cRNA standards were constructed exactly as described elsewhere made as previously described [63]. RNA concentrations were evaluated in all samples and quantified using a ND1000 spectrophotometer (Nanodrop). Real-time quantitative RT-PCR was performed with the TaqMan-RNA-to-CT 1-Step kit (Applied Biosystems/Life Technologies) in a 20 μL reaction mixture with 10 μL of 2x TaqMan RT-PCR Mix (containing ROX as a passive reference dye), 900 nM of forward and reverse primers, and 200 nM of FAM BHQ1-TP probe. Four μL of extracted RNA for supernatant samples and cRNA standards (serial dilutions), or 0.5 μg of total RNA for cell-associated (LA-N-5 cells infection) were used for the reaction. Amplification and detection were performed in a StepOnePlus Realtime PCR system apparatus and analysis were performed with the StepOne software version 2.3 (Applied Biosystems).

### Synthetic peptides and *in silico* cleavage assay

The synthetic peptides N-322: VDYSKNRRSRGAITTGY; sequence of rOC/ATCC reference virus S protein (amino acid 748–764) and N-321: VDYSKNRRSRRAITTGY; sequence of rOC/S_G758R_ virus S protein (amino acid 748–764) were made in-house at the laboratory of Dr. Robert Day. The underlined amino acid represents the G758R polymorphism between reference and clinical isolates. Briefly, recombinant PC enzymes were first titrated using the Dec-RVKR-chloromethylketone inhibitor. Cleavage assays of coronavirus derived peptides (42 μg/tube) were carried out with 5 nM of each PC with BSA in a final volume of 80 μl. The reaction was stopped with TFA (1% final). HPLC analysis (0–30% acetonitrile gradient, 0.5%/min) was done and quantification was obtained with peak area relative to T = 0 min. Peaks obatined were also collected and identified using MALDI-TOF.

### Statistical tests

For cell experiments, statistical analysis were conducted by one-way analysis of variance (ANOVA), followed by Tukey’s post hoc test, or a t-test. For mice experiments, results were compared using two non-parametric statistical tests: Kruskal-Wallis and Mann-Whitney. Survival rates were plotted as Kaplan–Meier survival curves and were compared using the log rank (Mantel–Cox) test. Statistical significance was defined as *p* < 0.05.

## Supporting Information

S1 Fig Astrogliosis is more important for rOC/ATCC compared to rOC/S_G758R_ in infected mouse brain after intranasal inoculation in 10 day-old BALB/c mice. Histological examination of astrogliosis and microgliosis in the brain. 10-day old BALB/c mice received 10^3.25^TCID_50_/10μL of rOC/ATCC, rOC/S_G758R_, or PBS by the IN route. Detection of glial fibrillary acidic protein (GFAP) in astrocytes in olfactory bulb (A) and in hippocampus (B) of infected mice at 9 dpi. (C) Detection of activated macrophages/microglia by an ascites fluid of the rat Mac-2 antibody in hippocampus of infected mice at 7 dpi. Magnification 200x.
(TIF)

S2 Fig Astrogliosis is more important for rOC/ATCC compared to rOC/S_G758R_ in infected mouse brain after intracerebral injection in 21 day-old BALB/c female mice. Histological examination of astrogliosis and microgliosis in the brain. 21-day old BALB/c mice received 10^2.5^TCID_50_/10μL of rOC/ATCC, rOC/S_G758R_, or PBS by the IC route. Detection of glial fibrillary acidic protein (GFAP) in olfactory bulb (A) or in hippocampus (B) of infected mice at 9 dpi. Detection of activated macrophages/microglia by an polyclonal anti-rabbit antibodies IBA-1 in the olfactory bulb (C) or in the hippocampus (D) of infected mice at 7 dpi. Magnification 200x.
(TIF)

S3 Fig Both variants are able to infect astrocytes as a secondary target in mixed primary CNS cultures from BALB/c mice at 24 hpi. Mixed primary cultures from BALB/c mice brain were infected with rOC/ATCC or rOC/S_G758R_ at MOI 0.1. Viral spread was evaluated at 8, 24, and 48 hpi. Astrocytes were stained in green with a mAb against a polyclonal rabbit anti-glial fibrillary acidic protein (GFAP) and the S viral glycoprotein in red. Results are representative of three independent experiments. Magnification 200x.
(TIF)

S4 Fig The S glycoprotein was cleaved at a second cleavage site. Overexposition of gels presented in Figs 6D and 7D. Western blot analysis of cell culture supernatant from mixed primary cultures from BALB/c mice brain (A) or differentiated human LA-N-5 cells (B) revealed the presence of an intermediate size fragment, S2’. Results are representative of three independent experiments.
(TIF)

S5 Fig Modulation of viral replication in a dose-dependent manner by dec-RVLR-cmk. The differentiated human neuroblastoma cell line (LA-N-5) was incubated only before infection or before and after infection with different concentration of furin-like inhibitor (dec-RVLR-cmk; 0, 5, 10, 20 and 40 μM). Infection was performed with rOC/ATCC or rOC/S_G758R_ at MOI 0.1. Kinetics of viral replication over a period of 48 h was evaluated. Titers of cell-associated virus for reference (A) or mutant virus (B), and free virus for reference (C) or mutant (D) virus were measured in cell and supernatant supplemented or not with dec-RVLR-cmk. (* P≤0.05). Results, shown in log_10_TCID_50_/mL are the mean values (with standard deviations) of three independent experiments.
(TIF)
