# Supplementary figures and images for: Cleavage of a Neuroinvasive Human Respiratory Virus Spike Glycoprotein by Proprotein Convertases Modulates Neurovirulence and Virus Spread within the Central Nervous System
Source: PLoS Pathog. 2015 Nov 6;11(11):e1005261. doi: 10.1371/journal.ppat.1005261 (PMC4636366; doi:10.1371/journal.ppat.1005261)

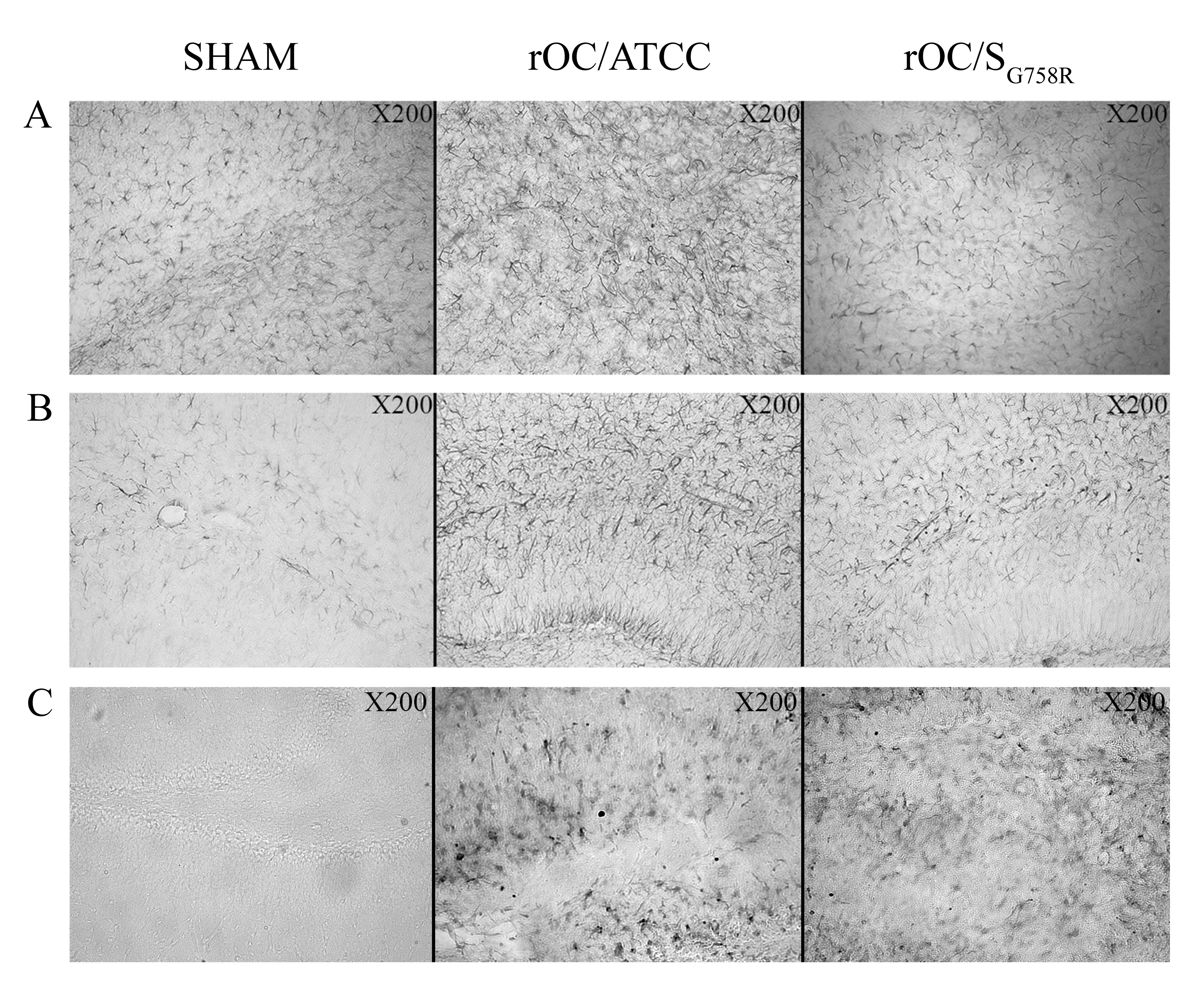

Supplement: S1 Fig — Histological examination of astrogliosis and microgliosis in the brain. 10-day old BALB/c mice received 103.25TCID50/10μL of rOC/ATCC, rOC/SG758R, or PBS by the IN route. Detection of glial fibrillary acidic protein (GFAP) in astrocytes in olfactory bulb (A) and in hippocampus (B) of infected mice at 9 dpi. (C) Detection of activated macrophages/microglia by an ascites fluid of the rat Mac-2 antibody in hippocampus of infected mice at 7 dpi. Magnification 200x. (TIF) [file ppat.1005261.s001.tif]

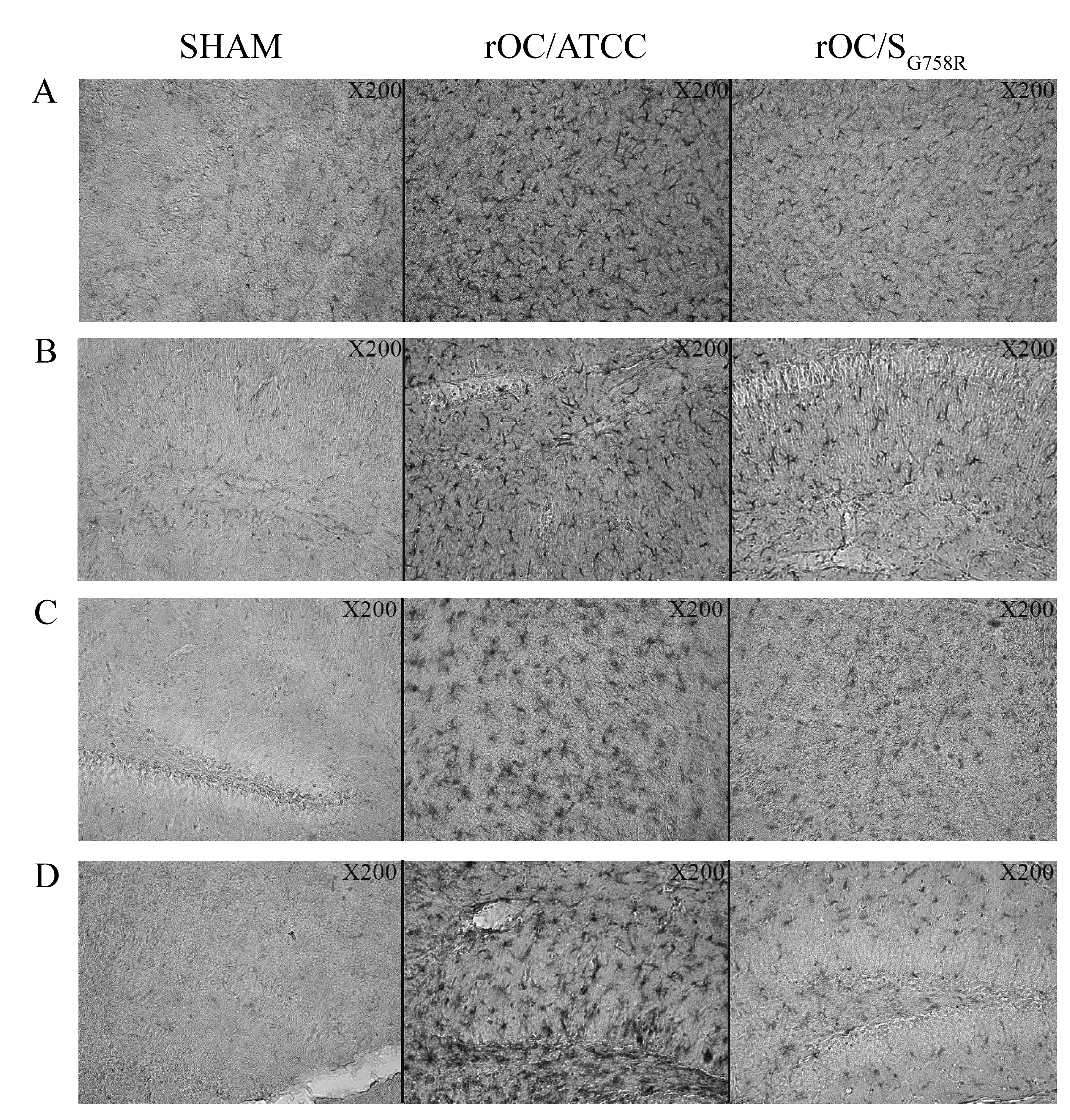

Supplement: S2 Fig — Histological examination of astrogliosis and microgliosis in the brain. 21-day old BALB/c mice received 102.5TCID50/10μL of rOC/ATCC, rOC/SG758R, or PBS by the IC route. Detection of glial fibrillary acidic protein (GFAP) in olfactory bulb (A) or in hippocampus (B) of infected mice at 9 dpi. Detection of activated macrophages/microglia by an polyclonal anti-rabbit antibodies IBA-1 in the olfactory bulb (C) or in the hippocampus (D) of infected mice at 7 dpi. Magnification 200x. (TIF) [file ppat.1005261.s002.tif]

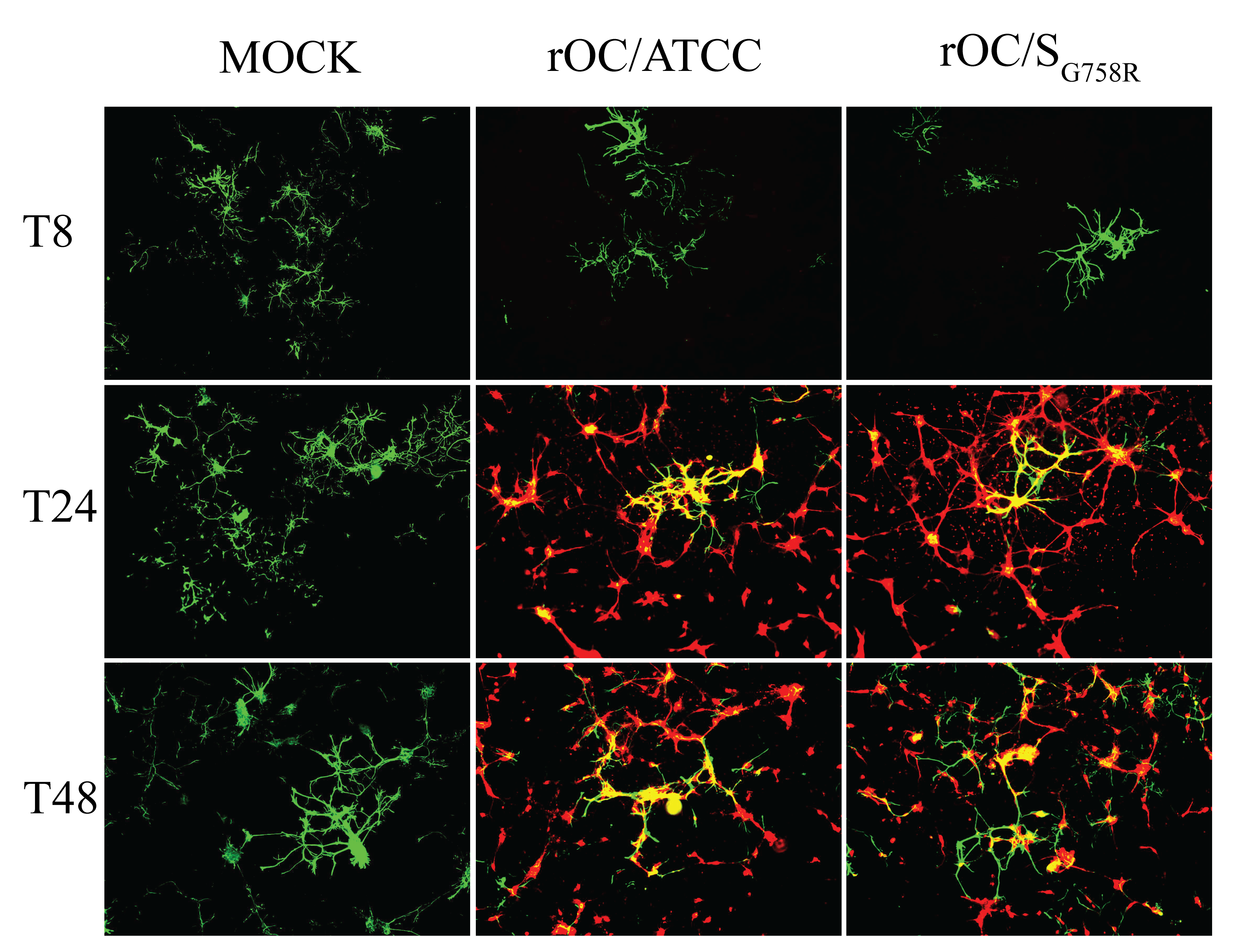

Supplement: S3 Fig — Mixed primary cultures from BALB/c mice brain were infected with rOC/ATCC or rOC/SG758R at MOI 0.1. Viral spread was evaluated at 8, 24, and 48 hpi. Astrocytes were stained in green with a mAb against a polyclonal rabbit anti-glial fibrillary acidic protein (GFAP) and the S viral glycoprotein in red. Results are representative of three independent experiments. Magnification 200x. (TIF) [file ppat.1005261.s003.tif]

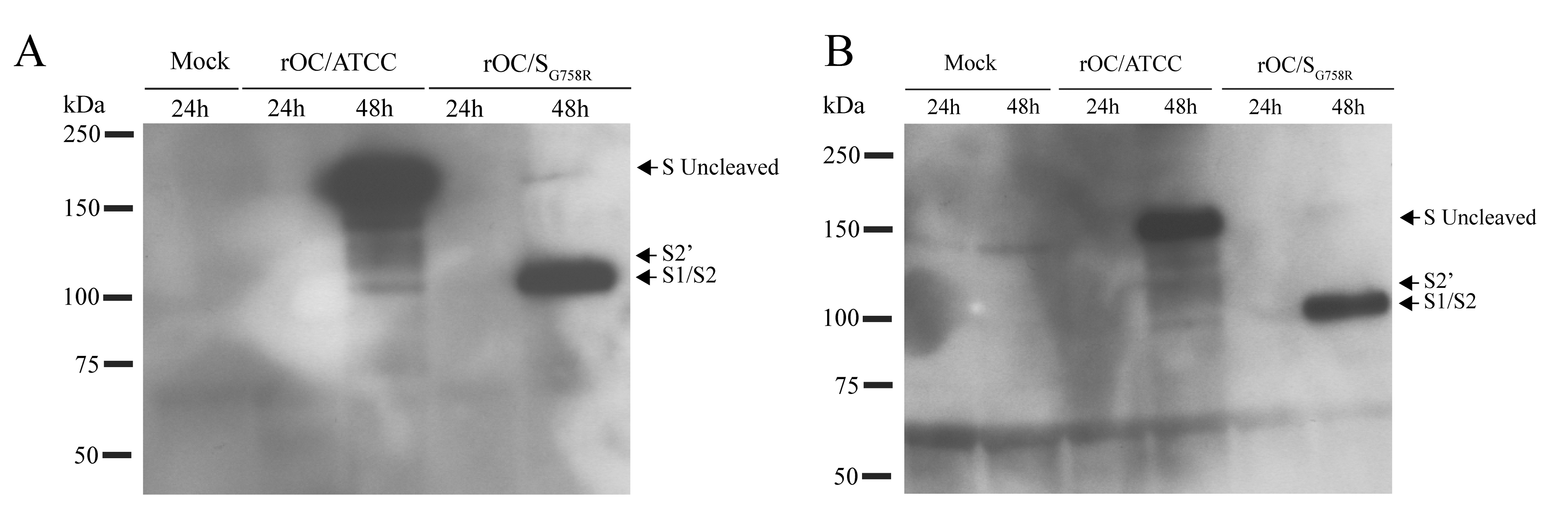

Supplement: S4 Fig — Overexposition of gels presented in Figs 6D and 7D. Western blot analysis of cell culture supernatant from mixed primary cultures from BALB/c mice brain (A) or differentiated human LA-N-5 cells (B) revealed the presence of an intermediate size fragment, S2’. Results are representative of three independent experiments. (TIF) [file ppat.1005261.s004.tif]

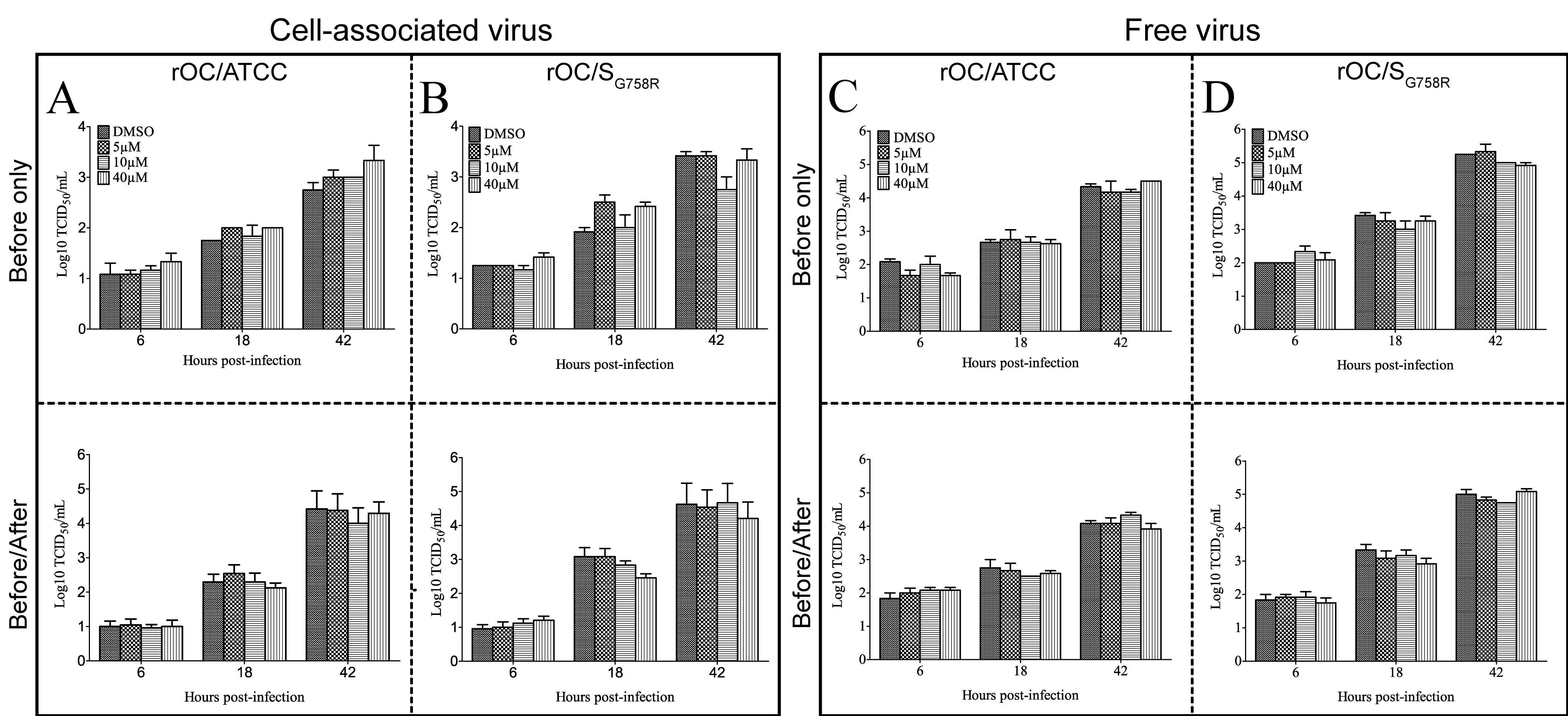

Supplement: S5 Fig — The differentiated human neuroblastoma cell line (LA-N-5) was incubated only before infection or before and after infection with different concentration of furin-like inhibitor (dec-RVLR-cmk; 0, 5, 10, 20 and 40 μM). Infection was performed with rOC/ATCC or rOC/SG758R at MOI 0.1. Kinetics of viral replication over a period of 48 h was evaluated. Titers of cell-associated virus for reference (A) or mutant virus (B), and free virus for reference (C) or mutant (D) virus were measured in cell and supernatant supplemented or not with dec-RVLR-cmk. (* P≤0.05). Results, shown in log10TCID50/mL are the mean values (with standard deviations) of three independent experiments. (TIF) [file ppat.1005261.s005.tif]
